# Characterization of a novel HIV-1 circulating recombinant form, CRF91_cpx, comprising CRF02_AG, G, J, and U, mostly among men who have sex with men

**DOI:** 10.1080/21505594.2022.2106021

**Published:** 2022-08-18

**Authors:** Cicek Topcu, Vasilis Georgiou, Johana Hezka Rodosthenous, Ioannis Demetriades, Brian Thomas Foley, Leondios G. Kostrikis

**Affiliations:** a Department of Biological Sciences, University of Cyprus, 1 University Avenue, Aglantzia, Nicosia, Cyprus; b AIDS Clinic, Larnaca General Hospital, Larnaca, Cyprus; c Los Alamos National Laboratory, T-6 Theoretical Biology and Biophysics, Los Alamos, NM, USA; d Cyprus Academy of Sciences, Letters, and Arts, Nicosia, Cyprus

**Keywords:** Human immunodeficiency virus type 1, circulating recombinant forms, unique recombinant forms, HIV-1 genetic diversity, HIV-1 phylogeny, HIV-1 molecular epidemiology, Cyprus

## Abstract

Prospective molecular studies of HIV-1 *pol* region (2253–5250 in HXB2 genome) sequences from sequenced samples of 269 HIV-1-infected patients in Cyprus (2017–2021) revealed a transmission cluster of 14 unknown HIV-1 recombinants that were not classified as previously established CRFs. The earliest recombinant was collected in September 2017, and the transmission cluster continued to grow until November 2020. Near full-length HIV-1 genome sequences of the 11 of the 14 recombinants were successfully obtained (790–8795 in HXB2 genome) and aligned against a reference dataset of HIV-1 subtypes and CRFs. We employed MEGAX for maximum-likelihood tree construction (GTR model, 1000 bootstrap replicates), Cluster-Picker for phylogenetic clustering analysis (genetic distance ≤0.045, bootstrap support value ≥70%), and REGA-3.0 for subtype determination. Bootscan and similarity plot analyses (sliding window of 400 nucleotides overlapped by 40 nucleotides) were conducted using SimPlot-v3.5.1, and subregion confirmatory neighbour-joining tree analyses were conducted using MEGAX (Kimura two-parameter model, 1000 bootstrap replicates, ≥70% bootstrap-support value). Exclusive clustering of the HIV-1 recombinants revealed their uniqueness. The recombination analyses illustrated the same unique mosaic pattern with six putative intersubtype recombination breakpoints, seven fragments of subtypes CRF02_AG, G, J and an unclassified fragment. We conclusively characterized the mosaic structure of the novel HIV-1 CRF, named CRF91_cpx, by the Los Alamos HIV Sequence Database. Additionally, we identified a URF of CRF91_cpx with two additional recombination sites, generated by a recombination event between subtype B and CRF91_cpx. Since the identification of CRF91_cpx, two additional patient samples have been entered into the CRF91_cpx transmission cluster, demonstrating active growth.

## Introduction

The human immunodeficiency virus type 1 (HIV-1) epidemic is an ongoing major health threat on a global scale. Despite the development and remarkable success of combination antiretroviral therapy (cART), which has led to considerable decreases in acquired immunodeficiency syndrome (AIDS)-related morbidity and mortality, HIV-1 transmission remains a major public health issue [1]. In 2020, 37.7 million people were living with HIV infection globally with 1.5 million them being newly infected [2]. According to the European Centre for Disease Prevention and Control (ECDC), the nationwide statistics demonstrate that in Cyprus there was an approximately 30% increase in the incidence of new HIV-1 infections in 2019 (n = 100) relative to 2018 (n = 78) and a 5% increase in 2020 (n = 105) relative to 2019 [3]. Thus, there is an evident increasing trend in new HIV-1 infections in Cyprus each year. A large-scale molecular epidemiology study including 336 HIV-1-infected patients and combining data from 1986 to 2012 demonstrated that the polyphyletic HIV-1 epidemic in Cyprus is driven by young Cypriot men who have sex with men (MSM) [4]. The preliminary results of a prospective molecular epidemiology study (C. Topcu *et al*., manuscript in preparation for publication) conducted from 2017 to 2021 show that the MSM community persists as the main driving force of HIV-1 transmission in Cyprus.

HIV-1 presents a high degree of genetic variability due to its elevated rate of molecular evolution. Consequently, HIV-1 has been divided into four groups (M, N, O and P), with ten distinct phylogenetic subtypes (A, B, C, D, F, G, H, J, K and L) within the major group, M [5,6]. In polyphyletic HIV-1 epidemics, the presence of multiple HIV-1 clades of HIV-1 group M subtypes in a population may lead to the coinfection of an individual with two or more HIV-1 clades. Coinfection accompanied by the highly recombinogenic nature of HIV-1 can potentially result in the generation of new HIV-1 recombinant strains [7]. An HIV-1 recombinant isolated from a single HIV-1-infected patient is classified as a unique recombinant form (URF) [8,9]. If an HIV-1 recombinant is isolated from three or more epidemiologically unlinked HIV-1-infected patients, given that the recombinants show an identical intersubtype mosaic structure, the HIV-1 recombinant is classified as a circulating recombinant form (CRF) [9,10].

The HIV-1 epidemic in Cyprus is highly polyphyletic as a consequence of the influx of various HIV-1 group M subtypes and CRFs from Africa, Europe, and Asia [4]. According to the most recent published study from Cyprus (2010–2012), the predominant subtype is subtype B (41%), followed by subtype A1 (19%) [11]. The presence of a number of CRFs has been reported (11%), among which CRF02_AG is predominant (4%). Although the polyphyletic HIV-1 epidemic persists in Cyprus, the preliminary results of the prospective molecular epidemiology study (2017–2021) (C. Topcu *et al*., manuscript in preparation for publication) suggest that subtype A1 is currently the predominant subtype (41%), followed by subtype B (33%). A comparison of the preliminary results with the last published data revealed a 10% increase in the prevalence of uncharacterized HIV-1 recombinants from 12% to approximately 22%, which could possibly be characterized as novel URFs or CRFs. The potential for the generation of novel URFs and CRFs arises in polyphyletic HIV-1 epidemics. In this context, CRF04_cpx was the first CRF that was characterized among HIV-1 isolates from Cyprus [12]. The CRF04_cpx strain was subsequently found in Greece [13]. Additionally, six URFs have been identified and characterized among HIV-1 isolates collected in Cyprus [14,15]. However, a number of URFs and CRFs remain to be assessed and characterized [11].

In this study, we describe the identification of a transmission cluster of HIV-1 recombinants that were not classified as previously established CRFs. Near full-length HIV-1 genome sequences of these HIV-1 recombinants were employed for phylogenetic and intersubtype recombination analyses to characterize their unique mosaic structure. The analyses performed as part of this study allowed the characterization of the novel CRF91_cpx circulating mostly among young Cypriot MSM reported to be infected in Cyprus. Additionally, the description of CRF91_cpx allowed the characterization of the CRF91_cpx strain URF. The results of our study underscore the increasing trend in the prevalence of novel URFs and CRFs, especially in regions with high HIV-1 genetic diversity. Moreover, our study highlights the importance of prospective molecular epidemiology studies for the routine supervision of HIV-1 transmission to provide clearer insights into the complexity of the HIV-1 epidemic.

## Materials and methods

### Study participants and sample requirements

The HIV-1 nucleotide sequences included in this study were isolated from 11 HIV-1-infected patients recruited as part of the prospective molecular epidemiology study (C. Topcu *et al*., manuscript in preparation for publication) conducted from 9 March 2017 to 14 October 2021 in Cyprus. All experimental protocols were approved by the Cyprus National Bioethics Committee (CNBC). Specifically, bioethical approval was obtained for the periods from 9 March 2017 to 13 October 2019 (approval number EEBK EΠ 2017.01.23, approval date 20 February 2017) and 14 October 2019 to 14 October 2021 (approval number EEBK ΕΠ 2019 71, approval date 2 September 2019). The inclusion criteria for the prospective molecular epidemiology study (2017–2021) (C. Topcu *et al*., manuscript in preparation for publication) was set based on the previously defined enrolment strategy [4,11,16]. Specifically, the inclusion criteria stated that all participating HIV-1-infected patients should be consenting, newly diagnosed or chronic patients who were antiretroviral naïve. Therefore, the study participants included in this study who were derived from the prospective molecular epidemiology study (C. Topcu *et al*., manuscript in preparation for publication) met the above inclusion criteria. This study collected data from the prospective molecular epidemiology study (C. Topcu *et al*., manuscript in preparation for publication) in accord with the relevant guidelines and regulations of the CNBC and the Office of the Commissioner for Personal Data Protection in Cyprus and with the written consent of all study participants. As such, written informed consent was obtained from all participating HIV-1-infected patients as part of the prospective molecular epidemiology study (C. Topcu *et al*., manuscript in preparation for publication), along with a questionnaire including detailed clinical, epidemiological and behavioural information, which was completed by qualified medical personnel. Moreover, the confidentiality of the study participants was ensured through double coding. As such, a unique hospital identification number, and subsequently, a unique laboratory identification number was assigned to each patient and the corresponding blood sample, respectively. The unique laboratory identification number cannot be traced back to the hospital identification number, which cannot be traced back to the name of the patient, enabling double coding. Accordingly, the samples used as part of this study were identified as CY467, CY494, CY520, CY533, CY614, CY622, and CY630 (collected during the period from 9 March 2017 to 13 October 2019 under the jurisdiction of bioethical approval EEBK EΠ 2017.01.23) and CY640, CY670, CY686, and CY742 (collected during the period from 14 October 2019 to 14 October 2021 under the jurisdiction of bioethical approval EEBK ΕΠ 2019 71). The blood samples, written informed consent of the HIV-1-infected patients, and the questionnaires containing corresponding detailed clinical, epidemiological and behavioural information were obtained at the Grigorios HIV Clinic of Larnaca General Hospital according to the guidelines and regulations of the CNBC. The Grigorios HIV Clinic of Larnaca General Hospital is the single clinical national service for the care of HIV-1-infected patients in Cyprus, providing a unique opportunity to obtain a representative cohort of the entire HIV-1 epidemic in Cyprus. The blood samples were later transferred to the Laboratory of Biotechnology and Molecular Virology of the University of Cyprus for processing.

### PCR amplification of HIV-1 pol region and sequencing

The blood samples were processed within two hours of sampling at the Laboratory of Biotechnology and Molecular Virology. The isolation of plasma from whole blood and subsequent HIV-1 RNA extraction from plasma using the QIAamp Viral RNA Mini Kit (QIAGEN, 52904) were performed according to previously described methodology and the manufacturer’s instructions [17]. As of 20 May 2021, blood samples from 277 HIV-1-infected patients had been collected as part of the prospective molecular epidemiology study (C. Topcu *et al*., manuscript in preparation for publication) conducted from 9 March 2017 to 14 October 2021. The *pol* (*protease, reverse transcriptase, integrase* and partial *vif*) region (2253–5250 in the HXB2 genome) sequences of the 269 HIV-1 viral genomes isolated from the 277 blood samples were successfully amplified according to a previously reported touchdown RT–PCR protocol, while the remaining eight samples were PCR negative [17]. The SuperScript™ IV One-Step RT–PCR System (ThermoFisher Scientific, 12594025) was used for primary RT–PCR, and Platinum™ Hot Start PCR 2× Master Mix (ThermoFisher Scientific, 13000012) was used for secondary nested PCR. A positive RNA control and a negative control were used in all PCR amplifications to confirm the success of the experiments and rule out the possibility of contamination, respectively. The sequencing of the 3259 bp final product of the pol RT–PCR assay employing 10 sequencing primers was conducted through Sanger sequencing by Macrogen Europe (https://dna.macrogen-europe.com/eng/) [17]. Finally, the HIV-1 genotypic subtypes based on the *pol* region nucleotide sequences were determined using the REGA HIV-1 Subtyping Tool Version 3.0 [18].

### Phylogenetic analyses of HIV-1 pol region nucleotide sequences

Next, a phylogenetic analysis of the obtained 269 HIV-1 *pol* region (2253–5250 in the HXB2 genome) nucleotide sequences was performed. Specifically, Molecular Evolutionary Genetics Analysis (MEGA X) software was utilized to generate a multiple sequence alignment using the ClustalW algorithm [19]. Thereafter, multiple sequence alignment was employed for the construction of a maximum likelihood phylogenetic tree in MEGA X software based on the general time-reversible (GTR) nucleotide substitution model with a gamma distribution. A total of 1,000 bootstrap replicates were used to assess the reliability of the phylogenetic clustering results. The maximum likelihood phylogenetic tree was then used to conduct phylogenetic clustering analysis using Cluster Picker software [20]. The parameters used in the phylogenetic clustering analysis, adopted from a previously published protocol, were as follows: 0.045 threshold of genetic distance and 70% bootstrap support value [4]. The phylogenetic maximum likelihood tree was visualized and edited using FigTree v1.4.3 software [21]. Phylogenetic clusters were classified as transmission clusters under the condition of a minimum of 3 patient samples clustering together based on previously established methodologies [4]. Through these analyses, a transmission cluster consisting of 14 HIV-1 recombinant sequences was identified among other molecular and transmission clusters. The HIV-1 genotypic subtypes of these 14 *pol* region nucleotide sequences were previously determined to represent unknown HIV-1 recombinant strains and were not classified as previously established CRFs, suggesting that these recombinants could belong to a newly emerging novel CRF strain.

### PCR amplification of near full-length HIV-1 genomes and sequencing

With the aim of identifying a possible newly emerging circulating recombinant form, near full-length HIV-1 genomes (790–8795 in the HXB2 genome) of the 14 HIV-1 recombinants encoding the *gag, pol, vif, vpr, tat, rev, vpu, env* and partial *nef* regions were amplified and sequenced using an in-house-developed near full-length HIV-1 genome RT–PCR assay (C. Topcu *et al*., manuscript in preparation for publication). This assay comprises three overlapping amplicons spanning the whole genome amplified via a previously described pol RT–PCR assay (2253–5250 in the HXB2 genome) [17], gag RT–PCR assay (790–2292 in the HXB2 genome) and env RT–PCR assay (5041–8795 in the HXB2 genome). The three PCR assays utilized HIV-1-specific primers targeting various HIV-1 group M subtypes, CRFs and recombinants. The SuperScript™ IV One-Step RT–PCR System (ThermoFisher Scientific, 12594025) was used for the primary RT–PCR assays of both the gag and env regions. For the secondary nested PCR assays of gag and env regions, Platinum™ Hot Start PCR 2× Master Mix (ThermoFisher Scientific, 13000012) and Platinum™ SuperFi II PCR Master Mix (ThermoFisher Scientific, 12368010) were used, respectively. For the sequencing of the final products of the three RT–PCR assays, we used a total of 29 sequencing primers to obtain the near full-length HIV-1 genome nucleotide sequences. Sanger sequencing was performed by Macrogen Europe (https://dna.macrogen-europe.com/eng/). Specifically, four sequencing primers were used for the sequencing of the 1721 bp final product of the gag RT–PCR assay, and 15 sequencing primers were used for the 4884 bp final product of the env RT–PCR assay. Then, the acquired sequencing primer amplicons were input into Geneious® 11.1.4 (https://www.geneious.com) software. When all amplicons obtained with the sequencing primers were composed, the mean number of aligned nucleotides per position was found to be approximately three, ranging from one to six at each nucleotide position. The quality of the sequencing readout and the success of each sequencing primer amplicon were defined as previously published [17]. Subsequently, the final HIV-1 gag and env region amplicons were aligned with the previously obtained HIV-1 pol region amplicons to obtain the consensus nucleotide sequences. The near full-length genomes of 11 of the 14 HIV-1 recombinants were successfully amplified and sequenced, while the remaining three HIV-1 recombinants failed the sequencing of the HIV-1 env region due to low-quality reads, and hence were not included in this study.

### Phylogenetic analyses of near full-length HIV-1 genome nucleotide sequences

The 11 near full-length HIV-1 genome nucleotide sequences (790–8795 in the HXB2 genome) were then used to repeat the phylogenetic analyses. The 11 HIV-1 recombinant sequences were aligned against a reference dataset of all known HIV-1 subtypes and CRFs using the RIP Alignment 2020 downloaded from the Los Alamos HIV Sequence Database (http://www.hiv.lanl.gov). The aforementioned methodology was repeated to perform phylogenetic analyses to demonstrate the exclusive phylogenetic clustering of these 11 HIV-1 recombinant sequences and, thus, prove their uniqueness. The multiple sequence alignment was created using MEGA X software (ClustalW algorithm), which was later employed for maximum likelihood phylogenetic tree construction (GTR model with a gamma distribution and 1,000 bootstrap replicates) [19]. The subsequent phylogenetic clustering analyses were conducted with Cluster Picker software (genetic distance of 0.045 as the threshold and 70% bootstrap support value) [20], and the resulting phylogenetic tree was visualized and edited with FigTree v1.4.3 software [21]. Consequently, the HIV-1 genotypic subtypes based on the near full-length HIV-1 genome nucleotide sequences were determined using the REGA HIV-1 Subtyping Tool Version 3.0 [18].

### Intersubtype recombination analyses

Detailed intersubtype recombination breakpoint analyses were carried out for the verification of recombination events. For the exploration of putative recombination patterns, bootscan and similarity plot analyses were performed on the near full-length HIV-1 genome nucleotide sequences in SimPlot v3.5.1 software [22]. These analyses were run against a reference dataset of HIV-1 group M subtypes (A, B, C, D, F, G, H, J and K) and CRF02_AG downloaded from the Los Alamos HIV Sequence Database (http://www.hiv.lanl.gov). Additionally, the reference dataset was enriched through Basic Local Alignment Search Tool (BLAST) analyses with the top two CRF02_AG BLAST hits. The BLAST analyses were performed with the HIV BLAST tool (https://www.hiv.lanl.gov/content/sequence/BASIC_BLAST/basic_blast.html) of the Los Alamos HIV Sequence Database using the default parameters. Primarily, the near full-length HIV-1 genome nucleotide sequences were aligned with the reference dataset in MEGA X software using the ClustalW algorithm [19]. Thereafter, for the identification and evaluation of putative intersubtype recombination breakpoints, bootscan and similarity plot analyses were performed in SimPlot v3.5.1 software. The parameters, adopted from a previously conducted study [14], were as follows: sliding window size of 400 nucleotides, overlapped by 40 nucleotides, with 1,000 bootstrap replicates. The identified putative intersubtype recombination breakpoints were confirmed with a jumping profile hidden Markov model (jpHMM) [23] and the Recombinant Identification Program (RIP) [24].

Subsequently, subregion confirmatory neighbour-joining tree analyses were carried out with MEGA X software for the further confirmation of intersubtype recombination breakpoints and the subtype origin of each fragment [19]. These analyses utilized the aforementioned reference dataset of HIV-1 group M subtypes (A, B, C, D, F, G, H, J and K) and CRF02_AG downloaded from the Los Alamos HIV Sequence Database (http://www.hiv.lanl.gov) as well as the top two CRF02_AG BLAST hits. The phylogenetic analyses employed the Kimura two-parameter nucleotide substitution model with 1,000 bootstrap replicates to assess the reliability of the phylogenetic clustering results. A neighbour-joining phylogenetic tree was constructed for each fragment of each HIV-1 recombinant query sequence. A bootstrap support value of 70% was considered to be definitive for the subtype origin. Due to relatively low bootstrap support values, the phylogenetic analyses of the last fragment were repeated based on the results of the BLAST analyses performed with the HIV BLAST tool (https://www.hiv.lanl.gov/content/sequence/BASIC_BLAST/basic_blast.html). Specifically, the neighbour-joining phylogenetic trees of the last fragments of all query sequences were reconstructed by excluding the reference sequences for subtype J and introducing reference sequences for CRF06_cpx. The reference sequences used for CRF06_cpx were the top three BLAST hits identified with the HIV BLAST tool (https://www.hiv.lanl.gov/content/sequence/BASIC_BLAST/basic_blast.html). Similarly, due to relatively low bootstrap support values, a maximum likelihood tree was constructed for the second fragment to confirm the results obtained from the neighbour-joining tree analyses. The maximum likelihood tree was constructed using the nucleotide sequences encoding the second fragments of all query sequences, the aforementioned reference dataset of HIV-1 group M subtypes, and numerous BLAST hits. The analyses were performed in IQ-TREE with a GTR-based substitution model and an SH-like approximate likelihood ratio test (SH-aLRT) for the estimation of branch support [25,26]. To summarize intersubtype mosaicism, the Recombinant HIV-1 Drawing Tool (https://www.hiv.lanl.gov/content/sequence/DRAW_CRF/recom_mapper.html) was used to generate mosaic genomic maps of the 11 HIV-1 recombinants. It is important to note that ten of the 11 HIV-1 recombinants showed the same mosaic pattern, while one of them presented a slightly different mosaic structure.

Consequently, for the exploration of other sequences with possible associations with the query sequences and similar intersubtype mosaicism, BLAST analyses were carried out with the HIV BLAST tool (https://www.hiv.lanl.gov/content/sequence/BASIC_BLAST/basic_blast.html). The ten HIV-1 recombinant sequences showing an identical mosaic structure were used as the query set. Each HIV-1 recombinant sequence was subjected to these comparative phylogenetic analyses separately, either as a near full-length genome sequence or as a partial sequence. The partial sequences were chosen with respect to the widely available HIV-1 regions in the database. In particular, the nucleotide sequences encoding the protease and reverse transcriptase proteins of the *pol* gene are the most common regions available in the database. These regions have been of great importance for drug resistance analyses, especially since the standardization of drug resistance testing in the early 2000s [27]. Nucleotide sequences of the V1V2 and V3 regions of the *env* gene are also widely available, as they have been regions of high interest for HIV vaccine development [28]. Upon the identification of other sequences showing possible associations with the query sequences, intersubtype recombination breakpoint analyses were performed. SimPlot v3.5.1 software was employed for the execution of bootscan and similarity plot analyses [22]. These analyses utilized the reference dataset of HIV-1 group M subtypes (A, B, C, D, F, G, H, J and K) downloaded from the Los Alamos HIV Sequence Database (http://www.hiv.lanl.gov) and included three BLAST hits for CRF02_AG. The bootscan and similarity plot analyses were subsequently repeated after the addition of the ten HIV-1 recombinant sequences to the reference dataset. This was followed by subregion confirmatory phylogenetic tree analyses, which were performed in IQ-TREE with a GTR-based substitution model and SH-aLRT branch support [25]. The previously mentioned reference dataset of HIV-1 group M subtypes (A, B, C, D, F, G, H, J and K) and CRF02_AG downloaded from the Los Alamos HIV Sequence Database (http://www.hiv.lanl.gov) were employed for these analyses.

## Data availability

All of the near full-length HIV-1 genome nucleotide sequences produced as part of this study have been deposited in the GenBank database, and the GenBank accession numbers are as follows: OK584018, OK283056, OK283057, OK283058, OK283059, OK283060, OK283061, OK283062, OK283063, OK283064 and OK283065.

The GenBank accession numbers of the sequences used in the reference dataset for the bootscan, similarity plot, and the subregion confirmatory phylogenetic tree analyses were as follows: A1-AF004885, A1-AF069670, A1-AF484509, A1-U51190; A2-AF286237, A2-GU201516, A2-AF286238; A3-AY521629; A4-AM000053; A6-AF413987; B-K03455, B-AY173951, B-AY423381, B-AY331295; C-AF067155, C-AY772699, C-U52953, C-U46016; D-U88824, D-K03454, D-AY253311, D-AY371157; G-U88826, G-AF061642, G-AF061641, G-AF084936; F1-AF077336, F1-AF005494, F1-AF075703, F1-AJ249238; F2-AF377956, F2-AJ249237, F2-AJ249236, F2-AY371158; J-AF082395, J-AF082394, J-GU237072; H-AF190128, H-AF005496, H-AF190127, H-FJ711703; K-AJ249235, K-AJ249239; 02_AG-L39106, 02_AG-AB485634, 02_AG-AF063223, 02_AG-AJ286994; 02_AG-AM279387, 02_AG-MN703132; 06_cpx-AJ245481, 06_cpx-AJ288982, 06_cpx-AJ288981.

The GenBank accession numbers of the top two hit sequences showing intersubtype mosaicism similar to CRF91_cpx identified through BLAST analyses were MH654876 and MH654941.

## Results

### Clinical, epidemiological and behavioural information of the study participants

We included 11 consenting HIV-1-infected patients living in Cyprus in this study. Their blood samples and corresponding patient questionnaires were collected at the Grigorios HIV Clinic of Larnaca General Hospital, according to the guidelines and regulations of the CNBC. The detailed clinical, epidemiological and behavioural information of the study participants obtained from the questionnaires is summarized in Table A1. Phylogenetic clusters were classified as transmission clusters under the condition of a minimum of 3 patient samples clustering together. As such, the collection dates of the first (CY467), second (CY494), and third (CY520) samples introduced into this HIV-1 recombinant transmission cluster were September 2017, January 2018, and May 2018, respectively. The collection date of the last sample introduced into the transmission cluster was November 2020. The dates of the first known positive HIV antibody tests of the cohort members ranged from June 2017 to September 2020. According to the information obtained, all 11 study participants were male (n = 11, 100%). The age of the study participants ranged from 29 to 75, with the most common age group being 26–35 (n = 4, 36%). The study participants mostly originated from Cyprus (n = 7, 64%), and the other study participants were from the UK (n = 2, 18%), Bulgaria (n = 1, 9%) and Nigeria (n = 1, 9%). Almost all the study participants reported being infected in Cyprus (n = 9, 82%), while one patient reported being infected in Nigeria (n = 1, 9%), and the country of infection for one patient was unknown (n = 1, 9%). The most common district of residence in the cohort was Pafos (n = 8, 73%), followed by Limassol (n = 2, 18%) and Larnaca (n = 1, 9%). Among the study participants, the most common route of infection was MSM (n = 7, 64%), followed by heterosexual contact (HC) (n = 2, 18%) and homo/bisexual contact (HBC) (n = 2, 18%). All the collected blood samples from the study participants had viral loads equal to or above 10^3^ RNA copies/mL plasma, while the lowest and the highest viral loads in this dataset were 1,590 RNA copies/mL plasma and 126,000 RNA copies/mL plasma, respectively. In addition, zero major drug resistance mutations and 21 accessory drug resistance mutations associated with non-nucleotide reverse transcriptase inhibitors (NNRTIs), protease inhibitors (PIs) or integrase strand transfer inhibitors (INSTIs) were identified. The following drug resistance mutations were identified: V179I (n = 3) associated with NNRTIs; K20I (n = 8) associated with PIs; and L74I/M (n = 9) and D232N (n = 1) associated with INSTIs. In line with the findings of the mutational analyses, all but one of the HIV-1 isolates were found to be susceptible to the antiretroviral drugs evaluated by the HIVdb Program of the Stanford University HIV Drug Resistance Database [29]. However, there was one HIV-1 isolate with potential low-level drug resistance to elvitegravir (EVG) and raltegravir (RAL).

### Phylogenetic analyses and HIV-1 genotypic subtypes

**Figure 1.**
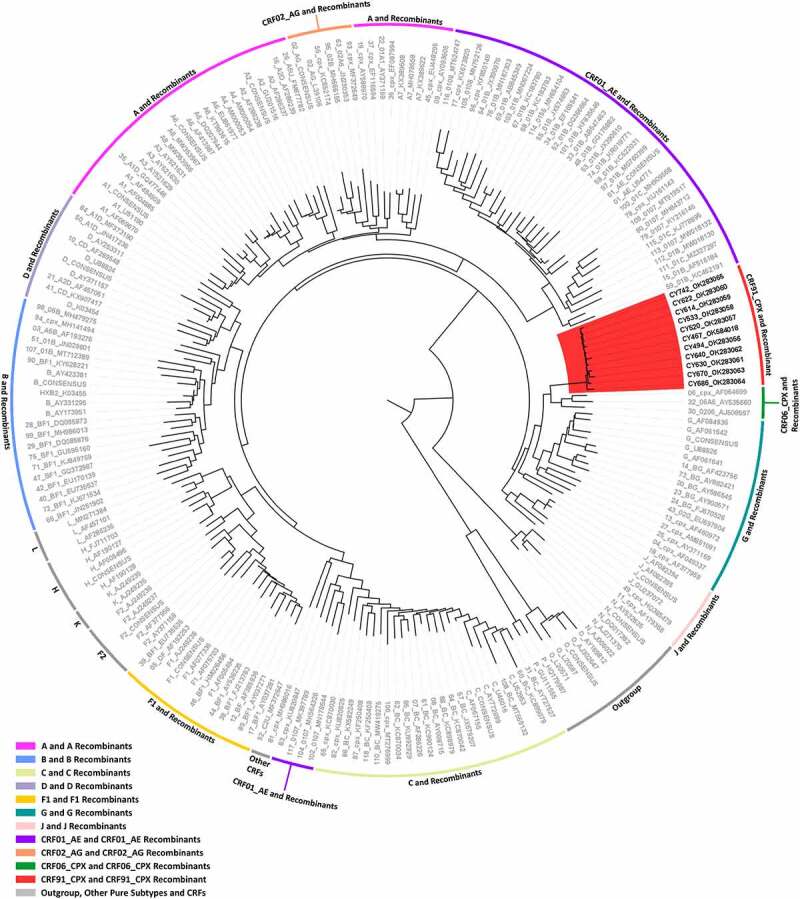
Maximum likelihood phylogenetic tree analyses of the near full-length genome sequences (790–8795 in the HXB2 genome) of 11 HIV-1 recombinants included in this study, derived from a cohort of consenting, newly diagnosed or chronic, antiretroviral naïve HIV-1-infected patients from Cyprus. The phylogenetic analyses were conducted against a reference dataset of all known HIV-1 subtypes and CRFs using the RIP Alignment 2020 downloaded from the Los Alamos HIV Sequence Database (http://www.hiv.lanl.gov). The HIV-1 clades are colour coded on the periphery of the maximum likelihood tree based on the HIV-1 genotypic subtypes, and the colour coding is defined below the tree. Each reference sequence is named to display the HIV-1 genotypic subtype, followed by the GenBank accession number, as indicated in grey. The sequences of the samples from Cyprus are indicated in black using their unique laboratory identification number, in which the prefix CY is followed by a number denoting the laboratory code. The branches highlighted in red represent the HIV-1 recombinant transmission cluster, consisting of the samples CY494, CY520, CY533, CY614, CY622, CY630, CY640, CY670, CY686, and CY742, characterized as CRF91_cpx. The sample CY467, which was characterized as the URF of CRF91_cpx, “Rec. of 91_cpx, B,” also appears within the HIV-1 recombinant transmission cluster highlighted in red. The phylogenetic clustering of the CRF91_cpx transmission cluster was considered to be definitive based on a genetic distance of 0.045 as the threshold and a 70% bootstrap support value.

As of 20 May 2021, blood samples from 277 HIV-1-infected patients had been collected as part of the prospective molecular epidemiology study (C. Topcu *et al*., manuscript in preparation for publication) conducted from 9 March 2017 to 14 October 2021. The HIV-1 *pol* region (2253–5250 in the HXB2 genome) was successfully amplified from 269 viral genomes isolated from the 277 blood samples. The remaining 8 patient samples were PCR negative. The phylogenetic analyses of the 269 HIV-1 *pol* region nucleotide sequences revealed a transmission cluster of 14 HIV-1 patient samples that were not classified as previously established CRFs. The earliest sample in this recombinant transmission cluster was collected in September 2017, and the cluster continued to grow until November 2020, when the most recent sample in this cluster was collected. The HIV-1 genotypic subtypes of these patient samples based on the *pol* region nucleotide sequences were previously identified as belonging to HIV-1 recombinant strains. The genotypic subtypes of these sequences were mostly defined as either “Rec. of 02_AG, G” or “Rec. of 02_AG, G, A1.” However, one sample was identified as “CRF02_AG-like” and one sample as “Rec. of G, A1, B.” Based on these results, the near full-length genomes of 11 of the 14 HIV-1 recombinants were successfully amplified and sequenced. In the remaining three HIV-1 recombinants, the sequencing of the HIV-1 env region (5041–8795 in the HXB2 genome) failed. Subsequent phylogenetic analyses of the near full-length HIV-1 genome sequences showed that the 11 HIV-1 recombinants did not cluster with any of the reference sequences of HIV-1 group M pure subtypes or previously established CRFs (RIP Alignment 2020) and instead exclusively clustered together with a high node support value of 1.0 (Figure 1). The HIV-1 recombinant transmission cluster formed next to the reference sequences of CRF06_cpx, CRF32_06A6 and CRF30_0206 with a low node support value of 0.645. The results of the phylogenetic analyses and the determined HIV-1 genotypic subtypes suggested that this cluster of HIV-1 recombinants may represent a novel CRF.

### Description of CRF91_cpx

**Figure 2.**
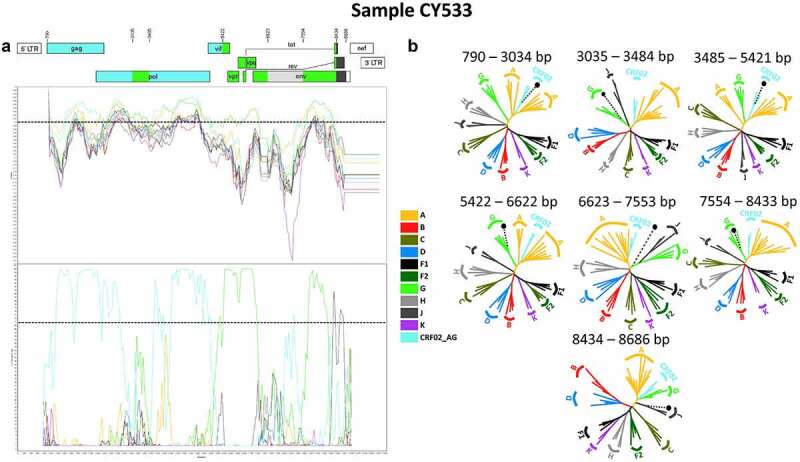
Recombination analyses of the near-full-length HIV-1 genome sequence (790–8795 in the HXB2 genome) of sample CY533 used as a representative sample to illustrate the intersubtype mosaic structure of the CRF91_cpx strain. The recombination analyses were conducted against a reference dataset of HIV-1 group M subtypes (A, B, C, D, F, G, H, J and K) and CRF02_AG downloaded from the Los Alamos HIV Sequence Database (http://www.hiv.lanl.gov) as well as the top two CRF02_AG BLAST hits. (A) The upper left diagram in this scheme illustrates the genomic map of sample CY533, which was generated by the Recombinant HIV-1 Drawing Tool (https://www.hiv.lanl.gov/content/sequence/DRAW_CRF/recom_mapper.html). The numbers above the diagram indicate the intersubtype recombination breakpoints in accordance with the HXB2 numbering. The near full-length HIV-1 genome was divided into seven fragments based on the six recombination breakpoints, showing its unique mosaic structure. The subtype origin of each fragment is colour coded in accord with informative analyses, and the colour coding is defined in the middle of the scheme. The middle left diagram displays the similarity plot analysis, in which the y-axis represents the percent similarity of the query sequence to the reference dataset. The bottom left diagram displays the bootscan analysis, where the y-axis represents the bootstrap support value. The x-axes of both diagrams represent the nucleotide positions in accordance with HXB2 numbering. The dotted horizontal line specifies the 70% bootstrap support value, which was considered to be definitive for subtype origin. The colour coding used for the similarity plot and bootscan analyses is identical to the colour coding of the genomic map. The similarity plot and bootscan analyses were performed in SimPlot v3.5.1 software. The parameters included a sliding window of 400 nucleotides, overlapped by 40 nucleotides, with 1,000 bootstrap replicates. (B) The right diagram illustrates the subregion confirmatory neighbour-joining tree analyses performed with MEGA X software. The neighbour-joining trees were constructed for each of the seven fragments characterized by the similarity plot and bootscan analyses. The phylogenetic analyses employed the Kimura two-parameter nucleotide substitution model with 1,000 bootstrap replicates to assess the reliability of the phylogenetic clustering results. A bootstrap support value of 70% was considered definitive for subtype origin. The region of the nucleotide sequences encoding each of the seven fragments is denoted above each tree with respect to HXB2 numbering. The dotted line ending with a black dot represents the query sequence of each tree. The colour coding used for the neighbour-joining trees is identical to the colour coding of the genomic map, similarity plot and bootscan analyses.

The near full-length HIV-1 genome nucleotide sequences were subjected to bootscan and similarity plot analyses, which confirmed the presence of a new set of recombination events that were not previously described. In addition, the HIV-1 genotypic subtypes based on the near full-length HIV-1 genome nucleotide sequences were previously identified as belonging to HIV-1 recombinant strains. Specifically, the HIV-1 genotypic subtypes of most of these sequences were defined as either “Rec. of 02_AG, G, J” or “Rec. of 02_AG, G, J, A1,” although one sample was identified as “Rec. of 02_AG, G, B, J, D.” These analyses illustrated that 10 of the 11 HIV-1 recombinants had the same and unique mosaic pattern, while one of them with the subtype “Rec. of 02_AG, G, B, J, D” had a slightly different mosaic structure. Each of the ten HIV-1 recombinant query sequences were composed of two CRF02_AG fragments, three subtype G fragments, one subtype J fragment and one fragment of unknown subtype origin. Figure 2(a) shows the mosaic genomic map and the bootscan and similarity plots for sample CY533, used as a representative sample for this recombinant transmission cluster. The figures illustrating the results of the rest of the HIV-1 recombinant samples are included in the Supplemental Material (Figure S1). The seven fragments were separated by six putative identical intersubtype recombination breakpoints, identified with SimPlot v3.5.1 software [22]. The six putative intersubtype recombination breakpoints (starting from the 5’ end) were located at nucleotide positions 3059 ± 24, 3448 ± 37, 5422, 6623, 7554 and 8434 (in the HXB2 genome) (Figure 2(a)). The putative intersubtype recombination breakpoints were confirmed via jpHMM [23] and RIP [24] analyses.

The genome of each HIV-1 recombinant was divided into seven fragments based on the identified putative intersubtype recombination breakpoints. Subregion confirmatory neighbour-joining tree analyses were performed on each fragment of each HIV-1 recombinant query sequence. These analyses further verified the intersubtype recombination breakpoints and the subtype origin of each fragment, as shown in Figure 2(b). The figures showing the results of the rest of the HIV-1 recombinant samples are included in the Supplemental Material (Figure S1). These phylogenetic analyses revealed that the first fragment (790–3034 or 790–3082 in the HXB2 genome) clustered with CRF02_AG with a 100% bootstrap support value. The second fragment (3035–3410 or 3035–3484 or 3083–3410 in the HXB2 genome) clustered with subtype G with bootstrap support values ranging from 72% to 78%. The third fragment (3411–5421 or 3485–5421 in the HXB2 genome) clustered with CRF02_AG with a 100% bootstrap support value. The fourth fragment (5422–6622 in the HXB2 genome) clustered with subtype G with a 100% bootstrap support value. The fifth fragment (6623–7553 in the HXB2 genome) did not cluster with any of the pure subtypes or CRFs. The sixth fragment (7554–8433 in the HXB2 genome) clustered with subtype G with bootstrap support values ranging from 99% to 100%. Finally, the seventh fragment (8434–8686 in the HXB2 genome) clustered with subtype J with bootstrap support values ranging from 70% to 84%, with one exception showing clustering at 59%. Regarding the lower bootstrap support values identified for the last fragment, BLAST analyses were performed for this fragment, which revealed higher genetic similarity of the last fragment to subtype J within the genome of CRF06_cpx. According to this information, the subregion confirmatory neighbour-joining tree analyses were repeated, and the results showed that the last fragment (8434–8686 in the HXB2 genome) clustered with CRF06_cpx with bootstrap support values ranging from 98% to 100%. A figure showing all the repeated neighbour-joining trees of the last fragment of each of the 11 HIV-1 recombinant samples is included in the Supplemental Material (Figure S2). As such, the mosaic structure of the ten HIV-1 recombinants was characterized and was designated CRF91_cpx by the Los Alamos HIV Sequence Database in accordance with the standards of HIV nomenclature (https://www.hiv.lanl.gov/content/sequence/HelpDocs/subtypes-more.html).

### Description of the CRF91_cpx URF

**Figure 3.**
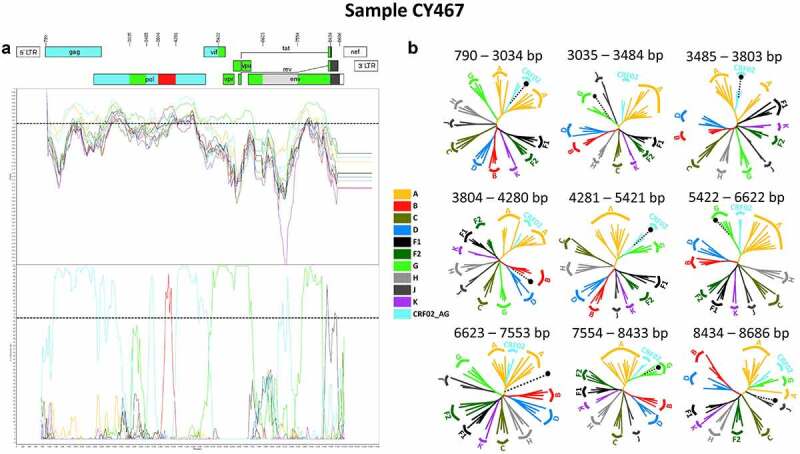
Recombination analyses of the near-full-length HIV-1 genome sequence (790–8795 in the HXB2 genome) of sample CY467 used to illustrate the intersubtype mosaic structure of the URF of the CRF91_cpx strain, “Rec. of 91_cpx, B.” The recombination analyses were conducted against a reference dataset of HIV-1 group M subtypes (A, B, C, D, F, G, H, J and K) and CRF02_AG downloaded from the Los Alamos HIV Sequence Database (http://www.hiv.lanl.gov) as well as the top two CRF02_AG BLAST hits. (A) The upper left diagram in this scheme illustrates the genomic map of sample CY467, which was generated by the Recombinant HIV-1 Drawing Tool (https://www.hiv.lanl.gov/content/sequence/DRAW_CRF/recom_mapper.html). The numbers above the diagram indicate the intersubtype recombination breakpoints in accordance with HXB2 numbering. The near full-length HIV-1 genome was divided into nine fragments based on the eight recombination breakpoints presenting its unique mosaic structure. The subtype origin of each fragment is colour coded in accord with informative analyses, and the colour coding is defined in the middle of the scheme. The middle left diagram displays the similarity plot analysis, where the y-axis represents the percent similarity of the query sequence to the reference dataset. The bottom left diagram displays the bootscan analysis, where the y-axis represents the bootstrap support value. The x-axes of both diagrams represent the nucleotide positions in accordance with HXB2 numbering. The dotted horizontal line specifies the 70% bootstrap support value, which was considered to be definitive for subtype origin. The colour coding used for the similarity plot and bootscan analyses is identical to the colour coding of the genomic map. The similarity plot and bootscan analyses were performed with SimPlot v3.5.1 software. The parameters included a sliding window of 400 nucleotides, overlapped by 40 nucleotides, with 1,000 bootstrap replicates. (B) The right diagram illustrates the subregion confirmatory neighbour-joining tree analyses performed with MEGA X software. The neighbour-joining trees were constructed for each of the nine fragments characterized by the similarity plot and bootscan analyses. The phylogenetic analyses employed the Kimura two-parameter nucleotide substitution model with 1,000 bootstrap replicates to assess the reliability of the phylogenetic clustering results. A bootstrap support value of 70% was considered to be definitive for subtype origin. The region of the nucleotide sequences encoding each of the nine fragments is denoted above each tree with respect to HXB2 numbering. The dotted line ending with a black dot represents the query sequence of each tree. The colour coding used for the neighbour-joining trees is identical to the colour coding of the genomic map, similarity plot and bootscan analyses.

The results of the bootscan and similarity plot analyses performed on near full-length HIV-1 genome nucleotide sequences revealed that one out of the 11 HIV-1 recombinants had a slightly different mosaic structure. The HIV-1 genotypic subtype of this recombinant was identified as “Rec. of 02_AG, G, B, J, D.” This HIV-1 recombinant query sequence was composed of three CRF02_AG fragments, three subtype G fragments, one subtype B fragment, one subtype J fragment and one fragment of unknown subtype origin. Figure 3(a) shows the mosaic genomic map and the bootscan and similarity plots for this HIV-1 recombinant, which belonged to sample CY467. The nine fragments were separated by eight putative intersubtype recombination breakpoints, identified in SimPlot v3.5.1 software [22]. The eight putative intersubtype recombination breakpoints (starting from the 5’ end) were located at nucleotide positions 3035, 3485, 3804, 4281, 5422, 6623, 7554 and 8434 (in the HXB2 genome) (Figure 3(a)). The putative intersubtype recombination breakpoints were confirmed by other software as described above. Sample CY467 shared the same composition of subtypes and intersubtype recombination breakpoints as CRF91_cpx, with two additional recombination sites caused by the recombination of subtype B into its genome within the third fragment (3485–5421 in the HXB2 genome) of CRF91_cpx. These results suggested that this sample was a URF of CRF91_cpx with subtype B.

The genome of the HIV-1 recombinants was divided into nine fragments based on the identified putative intersubtype recombination breakpoints, which were utilized in subregion confirmatory neighbour-joining tree analyses. The intersubtype recombination breakpoints and the subtype origin of each fragment were further verified, as shown in Figure 3(b). Based on the phylogenetic analyses, it was revealed that the first fragment (790–3034 in the HXB2 genome) and the second fragment (3035–3484 in the HXB2 genome) of the CRF91_cpx URF clustered with CRF02_AG and subtype G with bootstrap support values of 100% and 76%, respectively, displaying the same mosaic structure as CRF91_cpx. The third fragment (3485–3803 in the HXB2 genome) clustered with CRF02_AG with a 78% bootstrap support value. The fourth fragment (3804–4280 in the HXB2 genome) clustered with subtype B with an 89% bootstrap support value, demonstrating the main difference between CRF91_cpx and the URF of CRF91_cpx. Next, the fifth fragment (4281–5421 in the HXB2 genome) clustered with CRF02_AG with a 99% bootstrap support value. The rest of the genome of the URF of CRF91_cpx moving towards the 3’ end displayed the same mosaic structure as CRF91_cpx. Specifically, the sixth fragment (5422–6622 in the HXB2 genome) clustered with subtype G with a 100% bootstrap support value, while the seventh fragment (6623–7553 in the HXB2 genome) did not cluster with any of the pure subtypes or CRFs. The eighth fragment (7554–8433 in the HXB2 genome) clustered with subtype G with a 100% bootstrap support value. Finally, the ninth fragment (8434–8686 in the HXB2 genome) clustered with subtype J with a 79% bootstrap support value. As previously described, due to higher genetic similarity of the last fragment to subtype J within the genome of CRF06_cpx, as revealed by BLAST analyses, the subregion confirmatory neighbour-joining tree analyses were repeated for this region. The repeated phylogenetic analyses showed that the last fragment (8434–8686 in the HXB2 genome) clustered with CRF06_cpx with a 99% bootstrap support value, as shown in Figure S2 of the Supplemental Material.

### Identification of other CRF91_cpx sequences

The BLAST analyses carried out in the Los Alamos HIV Sequence Database revealed two partial sequences (2273–3869 in the HXB2 genome) with possible associations with the HIV-1 recombinant query sequences from Cyprus. These two sequences, MH654876 and MH654941, were the top two hits in the BLAST results, with 95% and 94% genetic similarity, respectively. Both samples were collected in Nigeria as part of a prospective observational cohort study focusing on MSM, and the collection dates were September 2015 for MH654876 and May 2014 for MH654941 [30]. The aligned partial sequences covered the 3’ end of the first fragment, the second fragment, and the 5’ end of the third fragment of the CRF91_cpx strain. The subtype assigned to these two partial sequences in the Los Alamos HIV Sequence Database (http://www.hiv.lanl.gov) indicated that they were recombinants of CRF02_AG and subtype G. This agrees with the genotypic subtype of the same region of the CRF91_cpx strain (2273–3869 in the HXB2 genome). The initial intersubtype recombination analyses performed with SimPlot v3.5.1 software [22] showed that the partial query sequences presented the same intersubtype mosaicism as the CRF91_cpx samples from Cyprus. Both query sequences comprised two CRF02_AG fragments and one subtype G fragment, as demonstrated in Figure S3 of the Supplemental Material. When the bootscan and similarity plot analyses were repeated after the addition of the ten CRF91_cpx sequences from Cyprus to the reference dataset, it was observed that the peak of CRF91_cpx was higher than the peaks of the reference sequences of the HIV-1 group M subtypes (A, B, C, D, F, G, H, J and K) and CRF02_AG. These results confirmed that the two query sequences had the same intersubtype mosaic structure and subtype origin as the CRF91_cpx samples from Cyprus (Figure S3). The three fragments were separated by the two putative intersubtype recombination breakpoints, as identified with SimPlot v3.5.1 software [22]. The two putative intersubtype recombination breakpoints (starting from the 5’ end) were located at nucleotide positions 3035 and 3485 (in the HXB2 genome) (Figure S3). These putative intersubtype recombination breakpoints were confirmed by jpHMM and RIP [24] analyses.

The genomes of the two query sequences were divided into three fragments based on the identified putative intersubtype recombination breakpoints. BLAST searches were performed on each of the three fragments of both query sequences separately for the further evaluation of the subtype origin of each fragment. Thereafter, subregion confirmatory phylogenetic tree analyses were performed on each fragment of the two query sequences, which further verified the intersubtype recombination breakpoints and the subtype origin of each fragment. The phylogenetic analyses for the two partial query sequences, MH654876 and MH654941, showed that the first fragments (2273–3034 in the HXB2 genome) clustered with CRF02_AG with 86% and 87% bootstrap support values, respectively. The second fragments (3035–3484 in the HXB2 genome) clustered with subtype G with an 81% bootstrap support value for MH654876 and 86% for MH654941. The third fragment (3485–3869 in the HXB2 genome) clustered with CRF02_AG with a 91% bootstrap support value for both query sequences.

## Discussion

In this study, using the near full-length genome sequences of ten HIV-1 recombinant viruses, we characterized a novel complex HIV-1 CRF in Cyprus, which was named CRF91_cpx by the Los Alamos HIV Sequence Database in accordance with the standards of the HIV nomenclature (https://www.hiv.lanl.gov/content/sequence/HelpDocs/subtypes-more.html). The ten HIV-1 recombinants shared the same unique intersubtype mosaic structure, which comprised seven fragments belonging to the CRF02_AG, G and J lineages, along with a fragment of unknown subtype origin that was not previously described. In fact, CRF91_cpx is the first CRF among the 118 CRFs reported in the Los Alamos HIV Sequence Database (https://www.hiv.lanl.gov/content/sequence/HIV/CRFs/crfs.comp), which resulted from a recombination event between parental viruses belonging to these three lineages. Our study also describes an HIV-1 recombinant of the newly characterized CRF91_cpx and subtype B, “Rec. of 91_cpx, B,” which was not previously described and was therefore identified as a URF. The study participants from whom the HIV-1 recombinant viruses were isolated showed no epidemiological links to each other and were living in three different cities in Cyprus: Pafos, Limassol and Larnaca. As such, the criteria set by the Los Alamos HIV Sequence Database for defining a new CRF (https://www.hiv.lanl.gov/content/sequence/HIV/CRFs/data/defining_new_CRFs.html) were met. It is noteworthy that Cyprus is experiencing a polyphyletic HIV-1 epidemic with an increasing trend of HIV-1 genetic diversity, as identified by previously conducted large-scale molecular epidemiology studies [4,11,14,15,16]. In this polyphyletic HIV-1 epidemic, CRF91_cpx is the second complex recombinant to be characterized among HIV-1 isolates collected in Cyprus, following the characterization of CRF04_cpx [12]. Moreover, six URFs have been identified and characterized among HIV-1 isolates collected in Cyprus thus far, along with four putative CRFs (C. Topcu *et al*., manuscript in preparation for publication) and numerous unknown URFs yet to be characterized [11,14,15]. The characterization of CRF91_cpx confirms the increasing prevalence of the generation and transmission of novel URFs and CRFs in these polyphyletic HIV-1 epidemics. This highlights the importance of prospective molecular epidemiology studies for the continuous surveillance of HIV-1 transmission and the necessity of near full-length HIV-1 genome analyses to better understand the complexity of the HIV-1 epidemic, especially in regions with high genetic diversity.

Specifically, the identification of a transmission cluster of 14 HIV-1 recombinants based on nucleotide sequences encoding the *pol* region of the HIV-1 genome was the initial indicator of a novel CRF. Hence, with the aim of characterizing the mosaic structure of these HIV-1 recombinants, we performed near full-length HIV-1 genome sequencing. The nucleotide sequences of the near full-length HIV-1 genomes were successfully amplified and sequenced for 11 out of 14 HIV-1 recombinants; in the remaining three HIV-1 recombinants, the sequencing of the HIV-1 env region (5041–8795 in the HXB2 genome) failed due to low-quality reads. It is possible that the failure in sequencing was caused by the high prevalence of secondary HIV-1 strains generated by frameshift mutations. The phylogenetic analyses performed on the nucleotide sequences of the near full-length HIV-1 genomes demonstrated that these sequences did not cluster with any of the reference sequences of HIV-1 group M pure subtypes or previously established CRFs. Therefore, the exclusive clustering of the HIV-1 recombinant nucleotide sequences with a high node support value of 1.0 strongly suggested that these sequences could possibly belong to a novel HIV-1 CRF lineage. Upon conducting detailed recombination analyses, it was established that CRF91_cpx comprised seven fragments separated by six intersubtype recombination breakpoints (starting from the 5’ end): 3059 ± 24, 3448 ± 37, 5422, 6623, 7554 and 8434 (in the HXB2 genome). The subtype origin was identified as CRF02_AG for two fragments, subtype G for three fragments and subtype J for one fragment, while the subtype origin of one of the fragments could not be identified. The subregion confirmatory neighbour-joining tree analyses illustrated that the fragments (starting from the 5’ end) clustered with CRF02_AG (100%), G (72–78%), CRF02_AG (100%), G (100%), U (subtype origin is unknown), G (99–100%) and J (59–84%). Despite the detailed analyses conducted, the fifth fragment of CRF91_cpx could not be characterized using the previously established HIV-1 group M subtypes or CRFs. Bootscan and similarity plot analyses were performed to investigate any associations between the fifth fragment of CRF91_cpx and the unclassified region of the previously characterized CRF04_cpx [12]. However, no associations could be identified; hence, the subtype origin of this fragment was concluded to be unclassified. The clustering of the second fragment (starting from the 5’ end) with subtype G with lower bootstrap support values (72–78%) indicated that this fragment was derived from subtype G, although it did not show high genetic similarity. Hence, a phylogenetic analysis was performed, and a maximum likelihood tree was constructed using the nucleotide sequences encoding the second fragment of CRF91_cpx along with numerous BLAST hits. The CRF91_cpx sequences and the BLAST hits, which were classified as subtype G, formed a clade in the phylogenetic tree. These analyses confirmed that the sequences belonged to the subtype G lineage. However, the CRF91_cpx sequences showed indications of divergence from the subtype G lineage, which could be attributed to the transmission and divergence of CRF91_cpx in the human population for some time. Nevertheless, the temporal evolutionary dynamics remain to be assessed. In conclusion, the second fragment was derived from a parental virus belonging to the subtype G lineage. Likewise, the last fragment clustered with subtype J with lower bootstrap support values (59–84%), suggesting that this fragment was derived from subtype J, although it did not show high genetic similarity. Hence, the subregion confirmatory neighbour-joining tree analyses were repeated by excluding the reference sequences of subtype J and introducing reference sequences of CRF06_cpx based on the BLAST results. These analyses revealed that the last fragment showed higher genetic similarity to CRF06_cpx, which was supported by high bootstrap support values (98–100%). Accordingly, this region of CRF06_cpx was also derived from subtype J; hence, it was concluded that the last fragment was derived from a parental virus belonging to the subtype J lineage. It should be noted that further investigation of the partial sequences (790–5250 in the HXB2 genome) of the remaining three HIV-1 recombinants, in which the sequencing of the HIV-1 env region failed, revealed them to show the abovementioned mosaic structure. Moreover, through BLAST analyses, we identified two partial sequences, MH654876 and MH654941, belonging to the CRF91_cpx strain. The recombination analyses showed the same mosaic structure, and the sequences were observed to closely cluster with the CRF91_cpx strain rather than the HIV-1 group M pure subtypes or CRFs. The comparison of the finalized mosaic structure of CRF91_cpx with the previously described CRFs showed that CRF91_cpx presented unique intersubtype mosaicism, which strongly confirmed that CRF91_cpx was not previously characterized.

Additionally, we identified and characterized a novel HIV-1 URF that emerged from a recombination event between CRF91_cpx and subtype B. The URF (CY467) showed higher genetic similarity to the CRF91_cpx samples than the other samples in the cohort of the prospective molecular epidemiology study (2017–2021) (C. Topcu *et al*., manuscript in preparation for publication). Hence, CY467 was added to the CRF91_cpx transmission cluster, which enabled its identification. Detailed recombination analyses established that CY467 had a slightly different mosaic structure than the CRF91_cpx strain. It comprised nine fragments separated by eight intersubtype recombination breakpoints (starting from the 5’ end): 3035, 3485, 3804, 4281, 5422, 6623, 7554 and 8434 (in the HXB2 genome). The subtype origin was identified as CRF02_AG for three fragments, subtype G for three fragments, subtype B for one fragment, and subtype J for one fragment, while the subtype origin of one of the fragments could not be identified. The subregion confirmatory neighbour-joining tree analyses illustrated that the fragments (starting from the 5’ end) clustered with CRF02_AG (100%), G (76%), CRF02_AG (78%), B (89%), CRF02_AG (99%), G (100%), U (subtype origin is unknown), G (100%) and J (79%). The comparison of the mosaic structure of CY467 with the newly characterized CRF91_cpx strain showed that CY467 shared the same composition of subtypes and intersubtype recombination breakpoints as CRF91_cpx, with two additional recombination sites (starting from the 5’ end): 3804 and 4281 (in the HXB2 genome). Therefore, it was concluded that the recombination of a parental virus belonging to the subtype B lineage into the genome of CRF91_cpx within the third fragment (3485–5421 in the HXB2 genome) resulted in the emergence of the URF of CRF91_cpx, designated “Rec. of 91_cpx, B.”

A total of 13 HIV-1 recombinants belonging to the CRF91_cpx strain and one HIV-1 recombinant characterized as the URF of the CRF91_cpx strain were identified among the 269 amplified HIV-1 nucleotide sequences. These sequences were isolated from 277 consenting, newly diagnosed or chronic, antiretroviral naïve HIV-1-infected patients who had been included in the prospective molecular epidemiology study (C. Topcu *et al*., manuscript in preparation for publication) as of 20 May 2021. However, it is crucial to highlight that between 20 May 2021 and the end date of the prospective molecular epidemiology study on 14 October 2021, an additional 36 blood samples were collected. The HIV-1 *pol* region (2253–5250 in the HXB2 genome) of the 36 HIV-1 viral genomes was successfully amplified and sequenced. Phylogenetic analyses revealed that two additional patient samples were introduced into the CRF91_cpx transmission cluster. The collection dates of these two samples were May 2021 and June 2021. Similar to the CRF91_cpx sequences used in this study, the HIV-1 genotypic subtypes of these two sequences were also identified as “Rec. of 02_AG, G” and “Rec. of 02_AG, G, A1” based on the analysis of the HIV-1 *pol* region. Furthermore, the preliminary analyses revealed the same mosaic structure in these two sequences that was observed in the CRF91_cpx strain. Henceforth, 15 samples of the CRF91_cpx strain were identified among the 305 HIV-1 viral sequences amplified. Thus, CRF91_cpx was estimated to comprise 4.9% (15/305) of the cohort of the prospective molecular epidemiology study (C. Topcu *et al*., manuscript in preparation for publication) conducted from 9 March 2017 to 14 October 2021. In accordance with the collection dates, the HIV-1 recombinant transmission cluster continued to grow until June 2021, since the first patient sample was introduced into this cluster in September 2017 (CY467), demonstrating the active growth of this cluster.

With respect to the clinical, epidemiological and behavioural information, it was established that the CRF91_cpx transmission cluster mostly consisted of young Cypriot MSM who reported being infected in Cyprus and resided in Pafos. The study participants were generally between the ages of 26 and 35; however, the age range of the cohort was significantly broad, ranging from 29 to 75. Despite the most common country of origin being Cyprus, the CRF91_cpx transmission cluster also included study participants from three other countries: the UK, Bulgaria and Nigeria. Although the results indicate that CRF91_cpx has circulated among the MSM community in Cyprus, the presence of two homo/bisexuals and two heterosexuals within the transmission cluster has important epidemiological implications and suggests that CRF91_cpx could possibly spread to a heterosexual network. As demonstrated by a previously conducted large-scale molecular epidemiology study (1986–2012) [4] and the preliminary results of the prospective molecular epidemiology study (2017–2021) (C. Topcu *et al*., manuscript in preparation for publication), the two epicentres of the HIV-1 epidemic are the urban regions of Nicosia and Limassol. The patients forming the transmission clusters mostly reside in these regions of Cyprus. Thus, it was observed that CRF91_cpx is mostly circulating away from the epicentre of the epidemic. However, within the CRF91_cpx transmission cluster, there were three patients residing in Limassol (n = 2) and Larnaca (n = 1). This suggests that the transmission of CRF91_cpx could potentially spread to the epicentre of the HIV-1 epidemic in the near future and result in increased growth of this cluster. Notably, the study participants mostly reported being infected in Cyprus, with one exception. One study participant from whom the URF of CRF91_cpx was isolated (CY467) was reported to be infected in Nigeria. The sample collected from this study participant was the earliest sample included in this recombinant transmission cluster, having been collected in September 2017. Accordingly, the two partial sequences of the CRF91_cpx strain identified through BLAST analyses, MH654876 and MH654941, were reported to be sampled from HIV-1-infected patients originating from Nigeria. The collection dates of MH654876 and MH654941 were recorded as September 2015 and May 2014, respectively, which implies that the CRF91_cpx strain has been circulating for some time. These observations suggest that CRF91_cpx was possibly imported to Cyprus from Nigeria. However, spatiotemporal evolutionary dynamics need to be assessed to better understand the place or time at which the CRF91_cpx strain originated. Nevertheless, the import of CRF91_cpx into Cyprus from Nigeria is highly plausible, as the preliminary results of the prospective molecular epidemiology study (2017–2021) (C. Topcu *et al*., manuscript in preparation for publication) show that approximately 4% of the cohort originated from Nigeria. Additionally, an estimated 17% of the cohort originated from Cameroon, which borders eastern Nigeria. According to a previously conducted large-scale molecular epidemiology study, gathering data from 1986 to 2012, 6% of the cohort originated from Eastern and Middle Africa, demonstrating an increased rate of migration to Cyprus [4]. The prevalence of the CRF91_cpx strain in Nigeria is challenging to evaluate, as there is an inadequate amount of molecular information. There are limited available sequencing data, and the available sequences are mostly partial sequences derived from molecular epidemiology studies focusing on drug resistance analyses. Previous studies showed that subtype G and CRF02_AG were the predominant HIV-1 strains in Nigeria, although the transmission of CRF06_cpx was also observed [30,31,32,33,34,35]. The co-circulation of these strains could have potentially resulted in the generation of the CRF91_cpx strain.

To reveal HIV-1 transmission dynamics in Cyprus in near real-time, we have developed and implemented a surveillance monitoring system that provides up-to-date visualization analytics. The visualization analytics were implemented on our laboratory website at https://www.kostrikislab.com/ongoing-projects/. In this surveillance monitoring system, detailed monthly phylogenetic clustering analyses are performed. As such, the samples received in the corresponding month are added to the dataset, and maximum likelihood phylogenetic trees are constructed. Monthly reports enable the continuous monitoring of the growth of existing transmission clusters and identification of newly created molecular and transmission clusters. Simultaneous analyses of clinical, epidemiological and behavioural information with molecular data allow the driving forces of HIV-1 transmission in Cyprus to be characterized. The most recent monthly phylogenetic report dates to September 2021 and comprises HIV-1 *pol* region (2273–3869 in HXB2 genome) nucleotide sequences derived from all HIV-1-infected patients received by our laboratory from 9 March 2017 to 14 October 2021 (Figure S4). The HIV-1-infected patients included in these monthly phylogenetic analyses are either newly diagnosed or chronic. The patients are included in these analyses regardless of their treatment status. However, in these analyses the first sample is included for each patient. Subsequent samples from the same patient (returning patient), are excluded from the analyses to prevent the inclusion of duplicate HIV-1 sequences isolated from the same HIV-1-infected patient. The most recent phylogenetic report presents the overall clustering data obtained since the implementation of this near real-time HIV-1 surveillance monitoring system. The active growth of the CRF91_cpx transmission cluster, with 16 patient samples clustering together, can be observed in Figure S4 of the Supplemental Material, as highlighted in red. Collectively, the CRF91_cpx transmission cluster is composed of 12 MSM, two HC and two HBC cases. Despite the current circulation of CRF91_cpx among the MSM community, the long-term inspection of the transmission of this newly characterized CRF is crucial for assessing the risk of spreading to a heterosexual network. Nonetheless, through these analyses, we identified an additional four transmission clusters comprising HIV-1 recombinants that were not classified as previously established CRFs. The HIV-1 genotypic subtypes assigned to these four transmission clusters based on *pol* region nucleotide sequences were “Rec. of A1, F1,” “Rec. of B, A1, D,” “Rec. of B, A1, F1” and “Rec. of B, A1, G.” The identified HIV-1 genotypic subtypes suggested that these transmission clusters of HIV-1 recombinants may lead to the characterization of four novel HIV-1 CRFs. Recombination analyses of these putative CRFs are currently being conducted. Moreover, we have identified two other phylogenetic clusters comprising HIV-1 recombinants that were not classified as previously established CRFs. The HIV-1 genotypic subtypes assigned to these two clusters based on the *pol* region nucleotide sequences were “Rec. of G, A1” and “Rec. of F2, G.” However, there are currently two patient samples clustered in both phylogenetic clusters. These phylogenetic clusters are under observation to monitor their growth. The introduction of one additional epidemiologically unlinked patient sample in each of the two HIV-1 recombinant clusters could suggest ongoing transmission of the two putative URFs, which may lead to the characterization of two additional novel HIV-1 CRFs.

Ultimately, we identified and characterized the novel CRF91_cpx, composed of CRF02_AG, G, J and an unclassified subtype, in Cyprus. The characterization of CRF91_cpx is relevant for molecular epidemiology studies for the identification of more HIV-1 sequences of the CRF91_cpx strain in other geographic areas, possibly in Eastern Africa. Our study highlights the significance of continuous surveillance through molecular epidemiology studies and analyses of near full-length or full-length HIV-1 sequences to evaluate the actual prevalence of HIV-1 recombinants and identify new CRFs and URFs. The continuous increase in HIV-1 genetic diversity and the frequent emergence of novel HIV-1 recombinant strains should receive our utmost attention. Thus, we should act upon the available data and make every effort to monitor and prevent the spread of HIV-1 infection.

## Supplementary Material

Supplemental Material

## Data Availability

The DNA nucleotide sequence data that support the findings of this study are openly available in GenBank at https://www.ncbi.nlm.nih.gov/genbank/

## Bioethics statement

Bioethical approval under approval number EEBK EΠ 2017.01.23 on February 20, 2017 was obtained for the period from March 9, 2017 to October 13, 2019, and bioethical approval under approval number EEBK ΕΠ 2019 71 on September 2, 2019 was obtained for the period from October 14, 2019 to October 14, 2021.

## Appendices

**Table A1.** Clinical, epidemiological and behavioural information of the 11 HIV-1-infected patients included in this study.

Patient^a^	Sex ^b^	Age Group (years)	Sample Collection Date	Positive Test Date ^c^	Country of origin ^d^	Risk group ^e^	CD4 (cells/mm^3^)	Plasma HIV-1 RNA (copies x 10^4^/ml)	Epidemiological Information ^f^	GenBank Accession Numbers
CY467	M	26-35	09/2017	06/2017	Nigeria	HBC	345	7,99	Infected in Nigeria	91B.CY.17.CY467.OK584018
CY494	M	26-35	01/2018	01/2018	Cyprus	HC	310	21,70	Infected in Cyprus	91_CPX.CY.18.CY494.OK283056
CY520	M	46-55	05/2018	03/2018	Cyprus	MSM	300	28,70	Infected in Cyprus	91_CPX.CY.18.CY520.OK283057
CY533	M	26-35	07/2018	05/2018	Cyprus	HC	851	1,59	Infected in Cyprus	91_CPX.CY.18.CY533.OK283058
CY614	M	26-35	06/2019	04/2019	Cyprus	MSM	237	126,00	Infected in Cyprus	91_CPX.CY.19.CY614.OK283059
CY622	M	66-75	07/2019	05/2019	UK	MSM	694	8,78	N/A	91_CPX.CY.19.CY622.OK283060
CY630	M	66-75	09/2019	-/2019	Cyprus	MSM	294	32,40	Infected in Cyprus	91_CPX.CY.19.CY630.OK283061
CY640	M	36-45	10/2019	09/2019	Cyprus	MSM	426	31,70	Infected in Cyprus	91_CPX.CY.19.CY640.OK283062
CY670	M	56-65	02/2020	01/2020	Bulgaria	MSM	103	46,30	Infected in Cyprus	91_CPX.CY.20.CY670.OK283063
CY686	M	56-65	05/2020	05/2020	UK	MSM	131	107,00	Infected in Cyprus	91_CPX.CY.20.CY686.OK283064
CY742	M	46-55	11/2020	09/2020	Cyprus	HBC	309	86,40	Infected in Cyprus	91_CPX.CY.20.CY742.OK283065

^a^Indicates the laboratory code for each study participant. ^b^ M, male. ^c^ Indicates the date (month/year; -, unknown month) of the first known positive HIV antibody test. ^d^ Country of birth of the study participants; UK, United Kingdom. ^e^ HBC, homo/bisexual contact; HC, heterosexual contact; MSM, men who have sex with men. f Information provided by the study participants. N/A, Not available.
