# Supplementary material for: Characterization of a novel HIV-1 circulating recombinant form, CRF91_cpx, comprising CRF02_AG, G, J, and U, mostly among men who have sex with men
Source: Virulence. 2022 Aug 18;13(1):1331–48. doi: 10.1080/21505594.2022.2106021 (PMC9397478; doi:10.1080/21505594.2022.2106021)
Supplement: Supplemental Material [file KVIR_A_2106021_SM9112.docx]

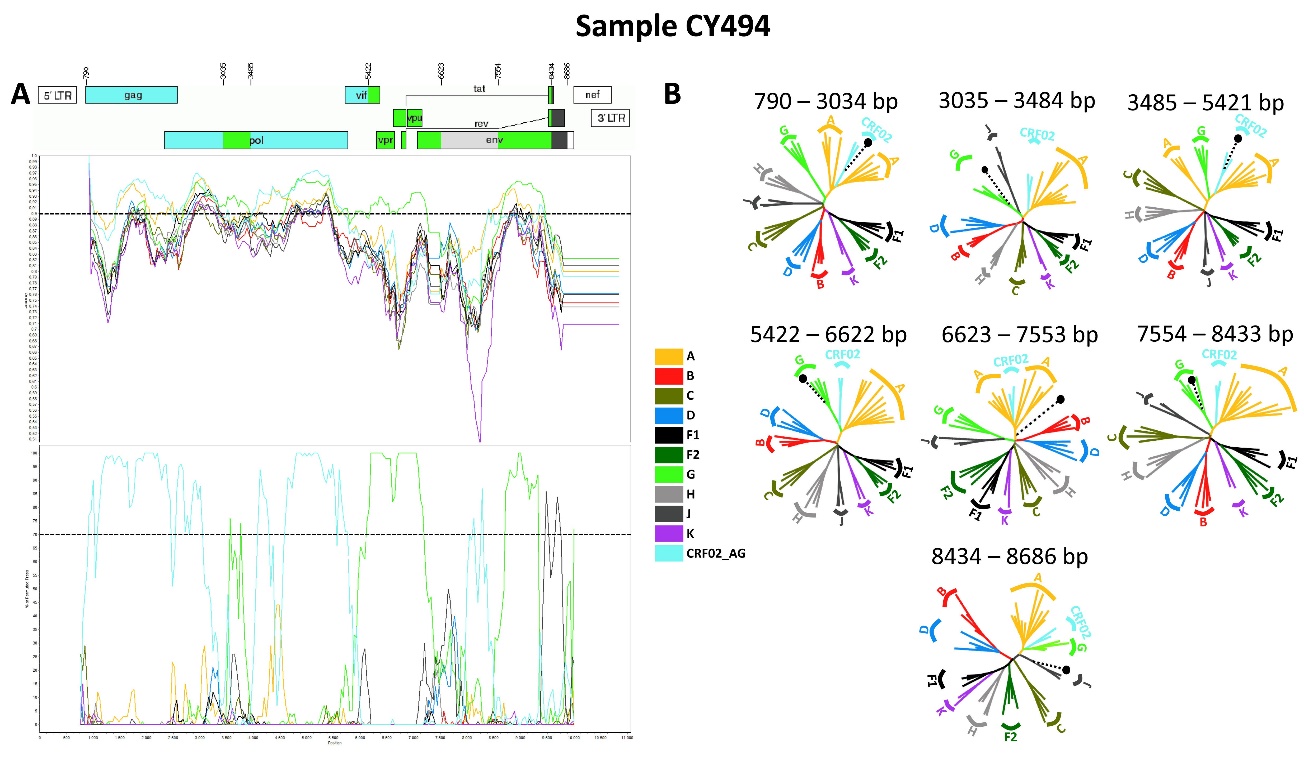

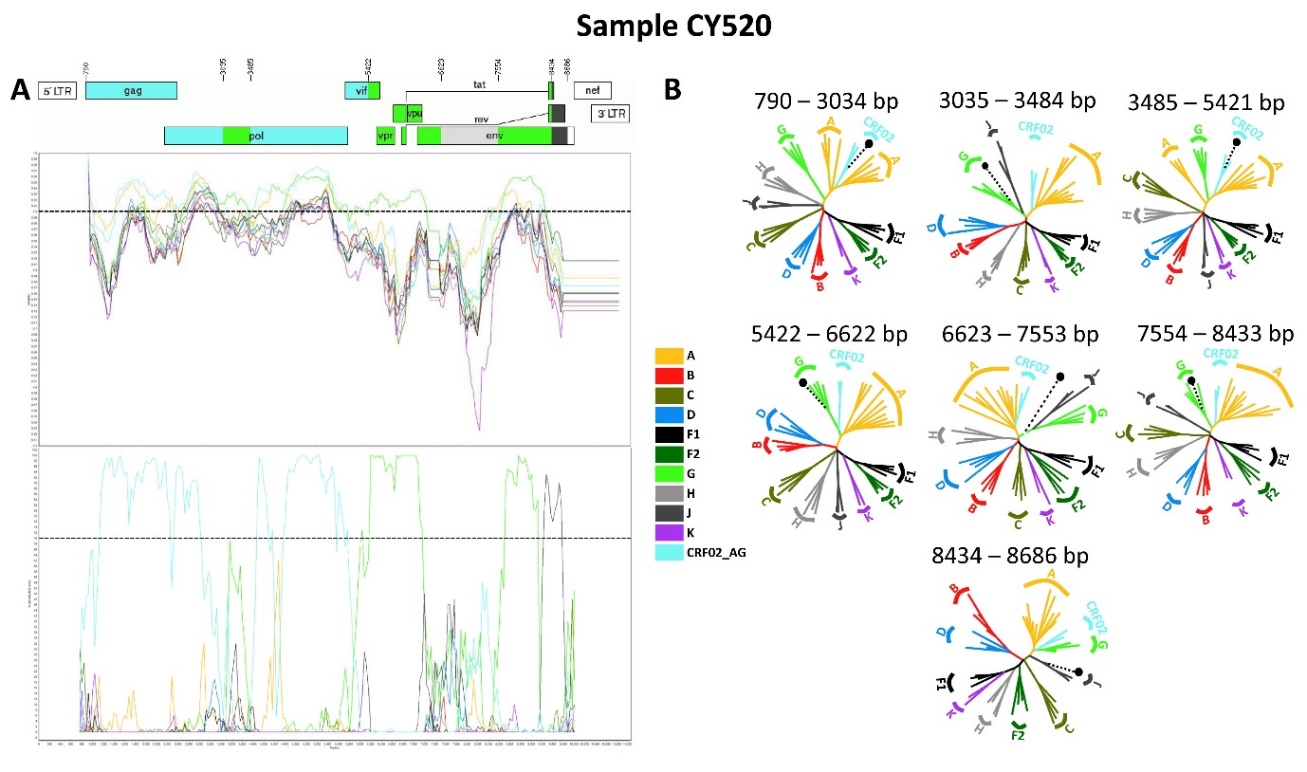

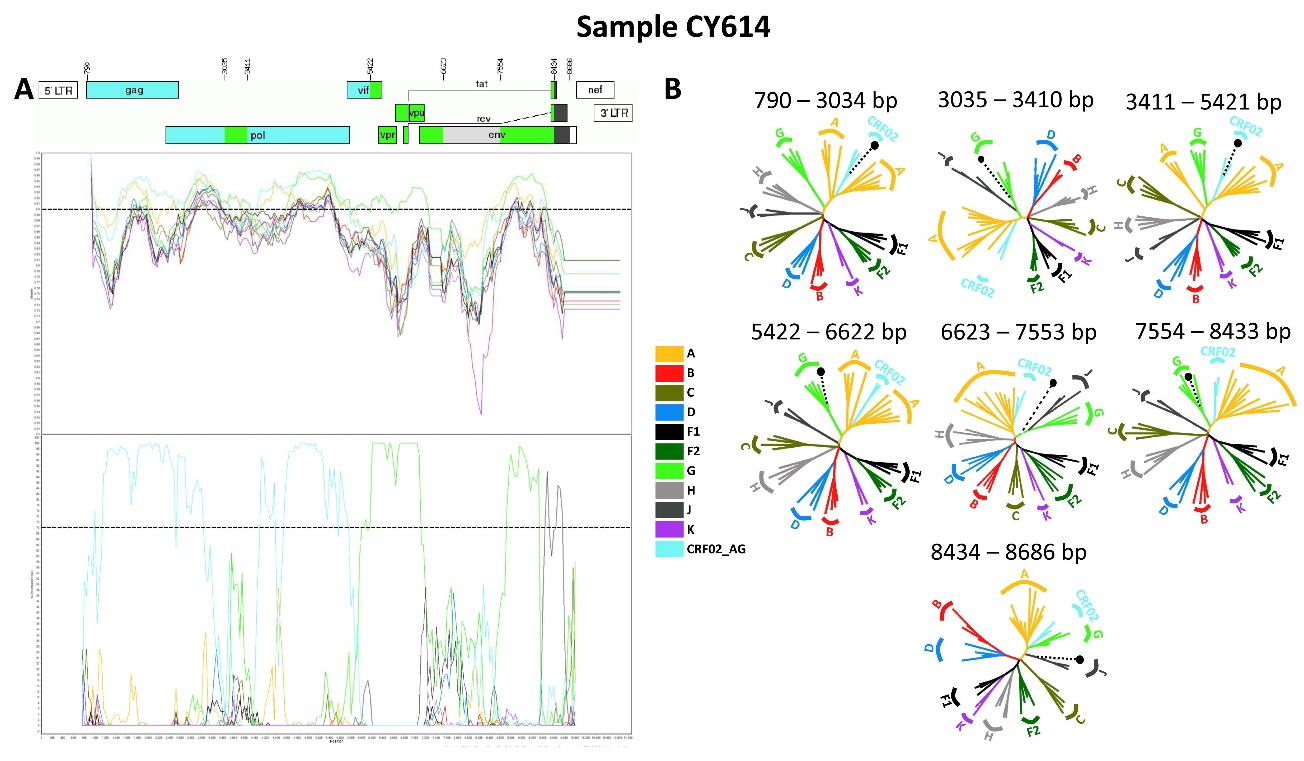

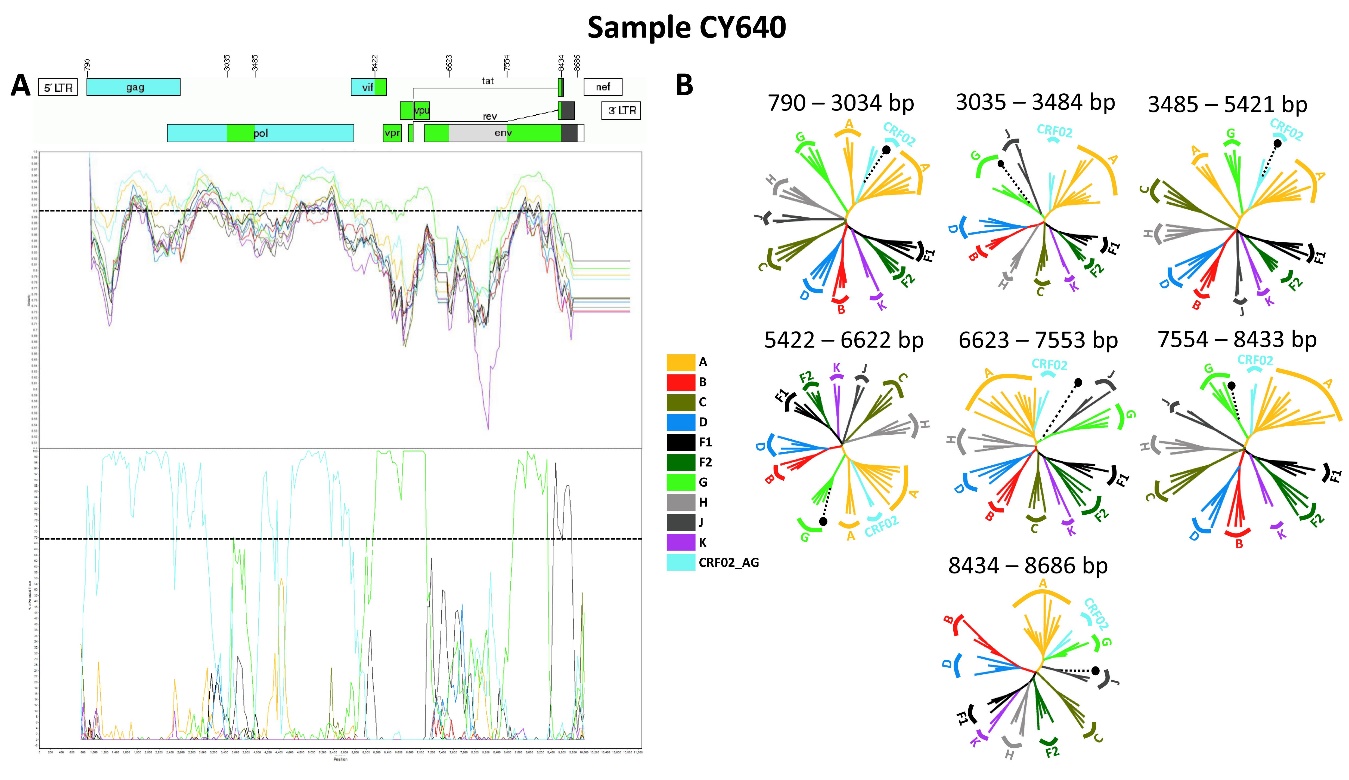

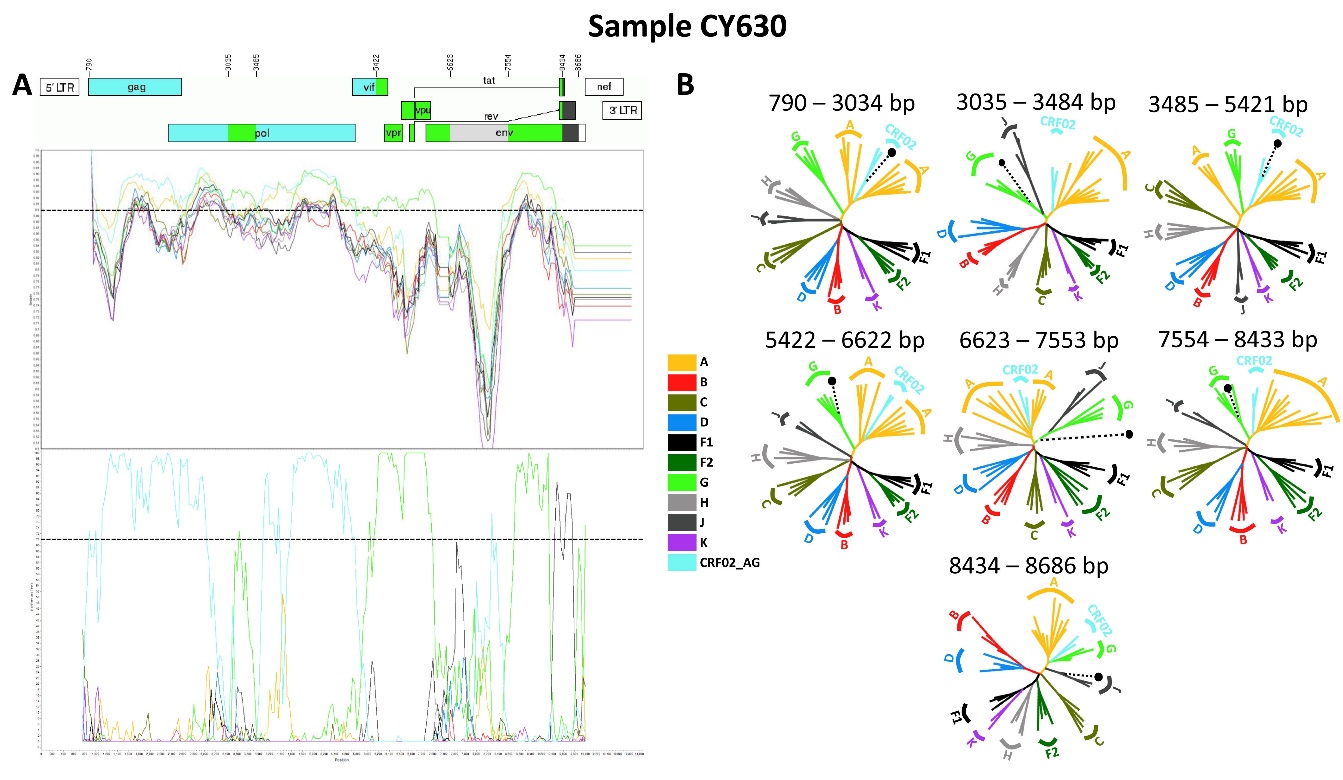

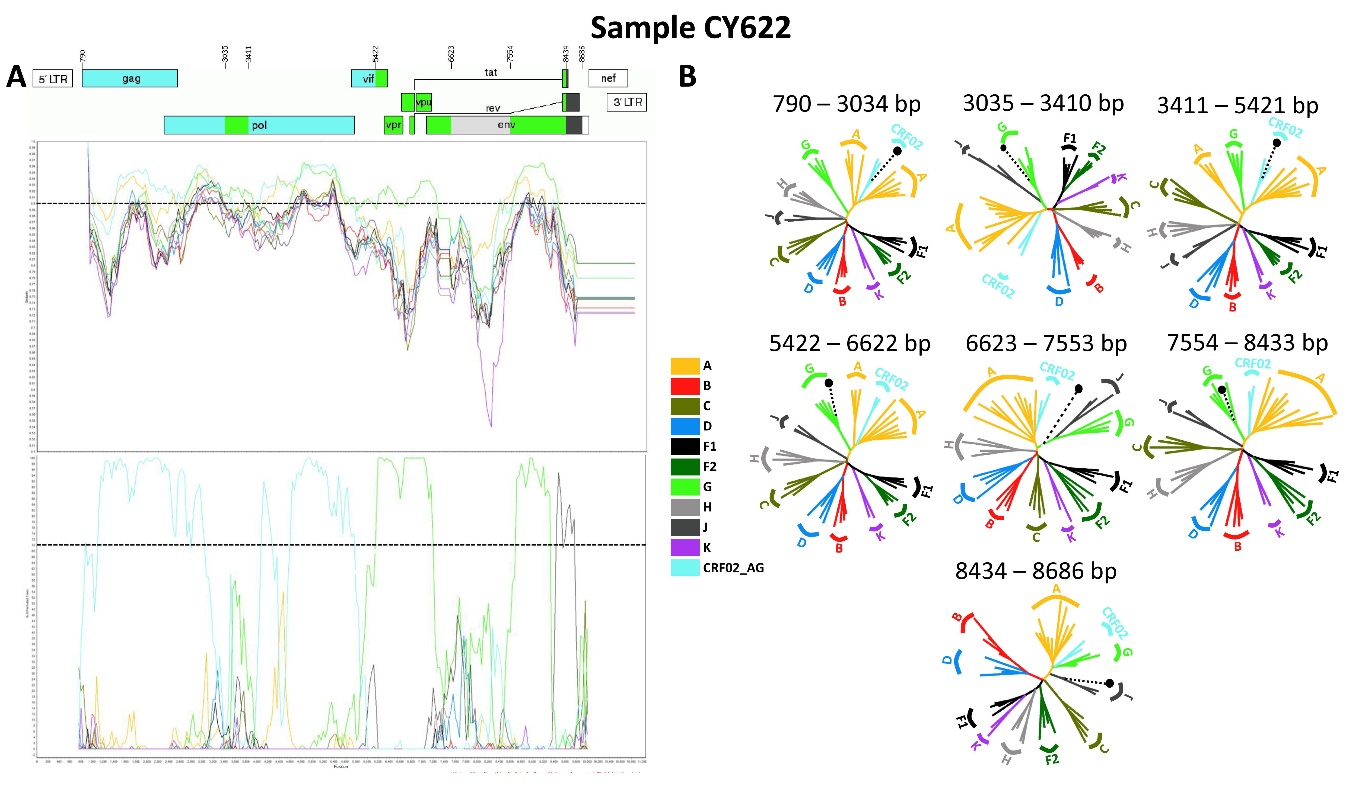

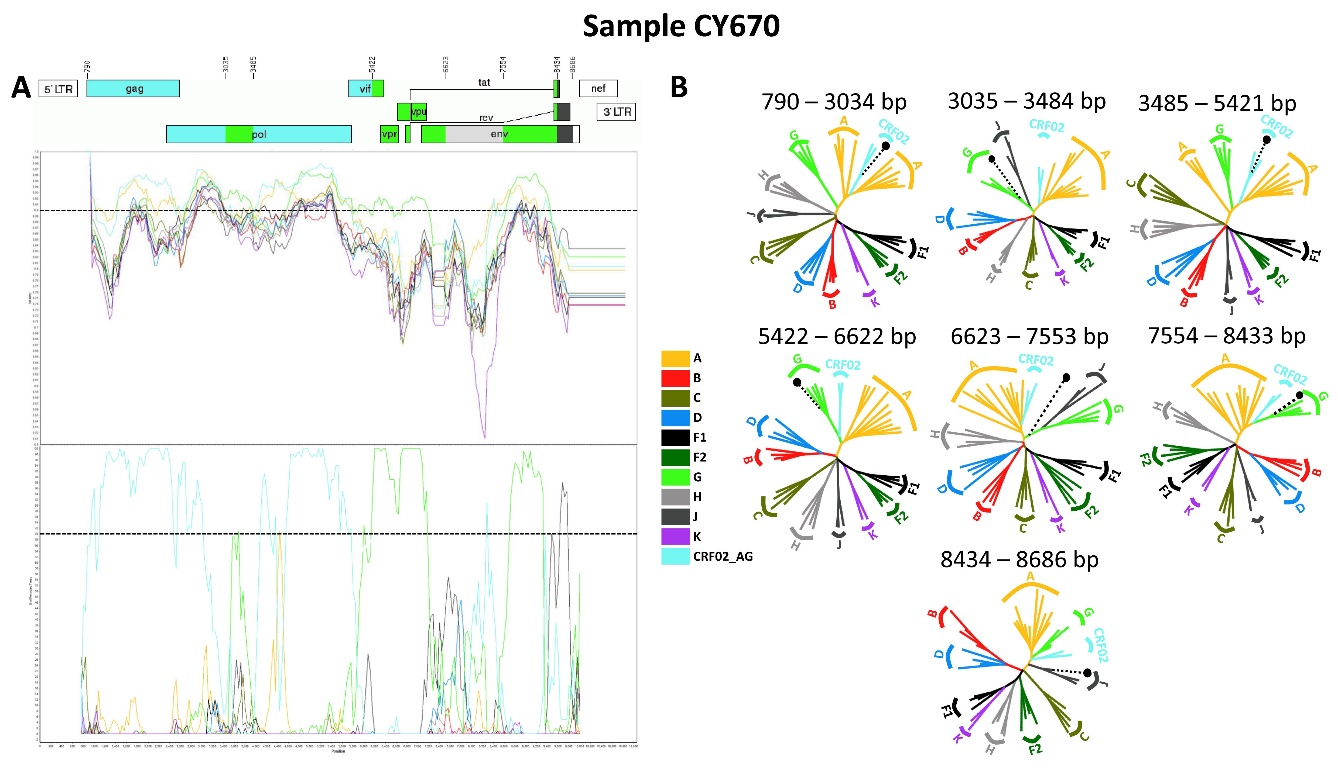

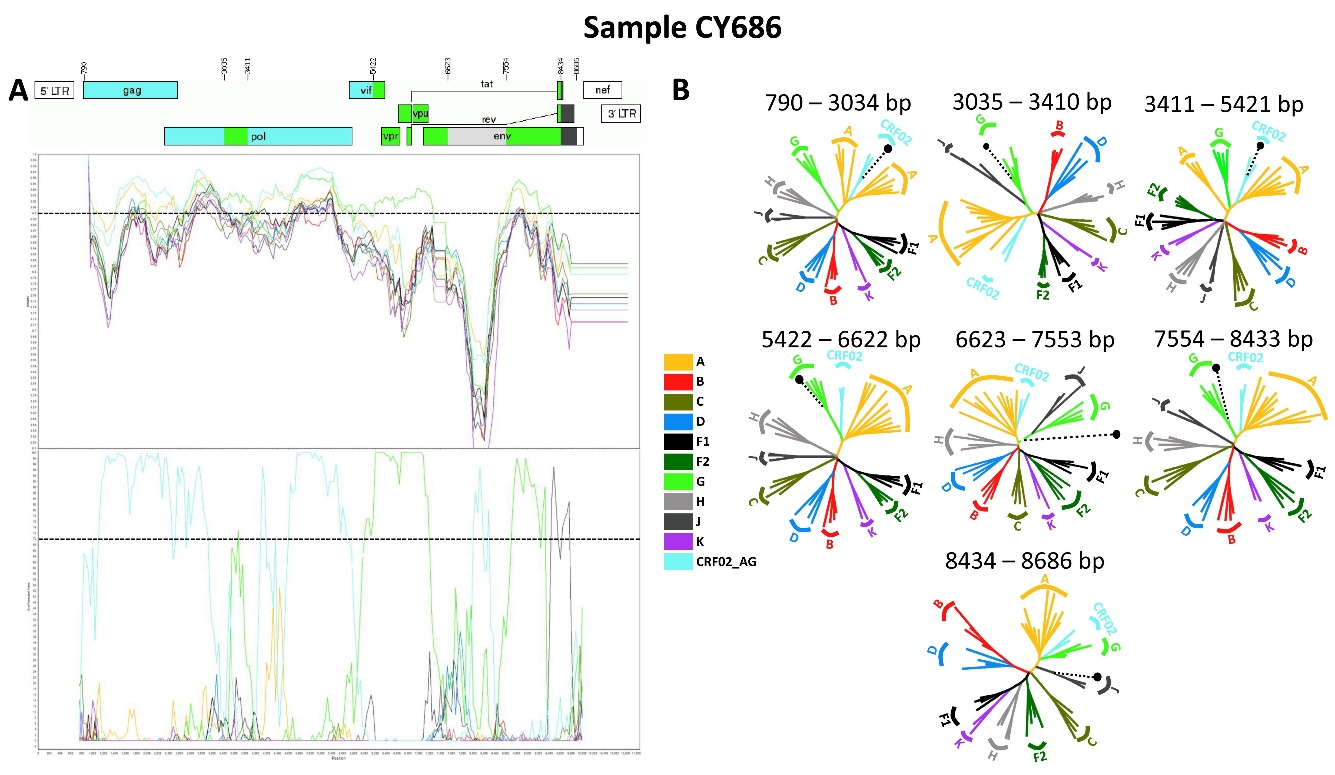

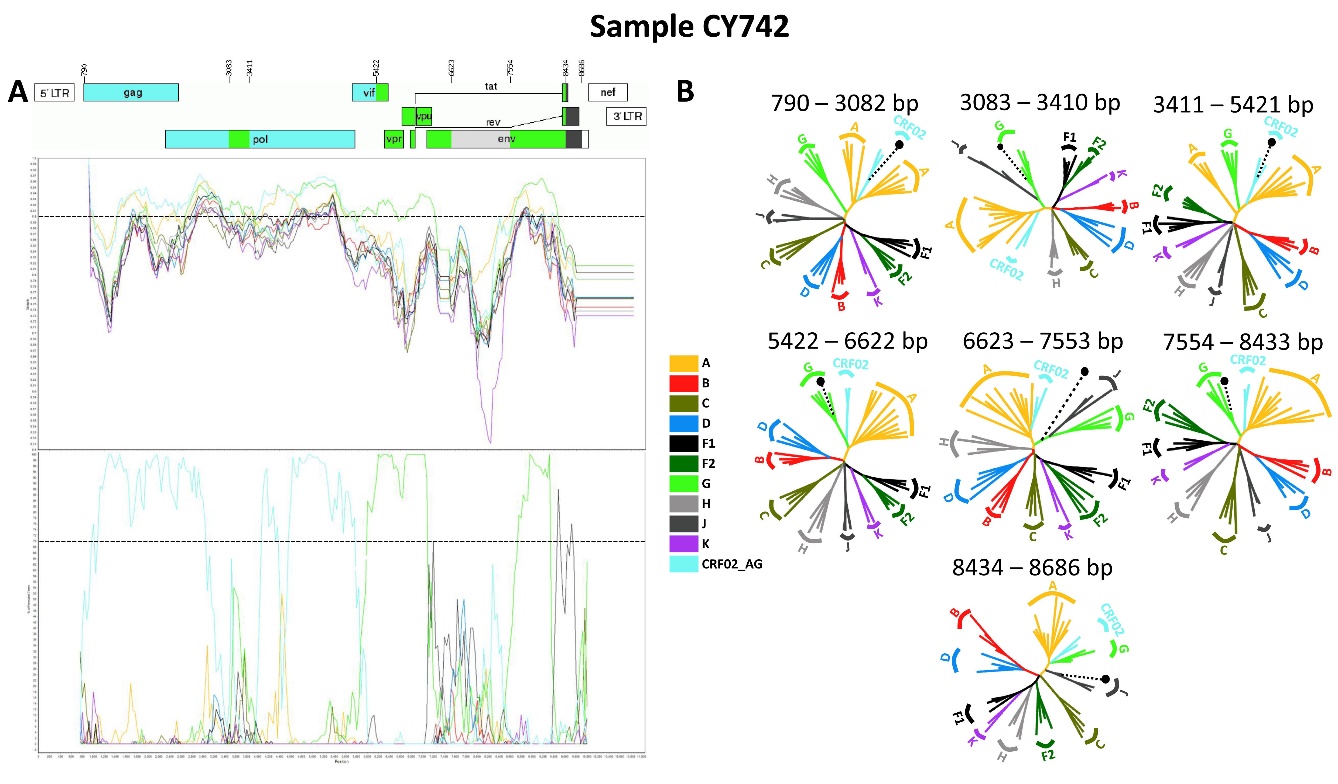


**Figure S1.** Recombination analyses of the nine near-full-length HIV-1 genome sequences (790-8795 in the HXB2 genome) derived from samples CY494, CY520, CY614, CY622, CY630, CY640, CY670, CY686 and CY742, illustrating the intersubtype mosaic structure of the CRF91_cpx strain. Each scheme in this figure represents each of the aforementioned samples, as indicated above each scheme. The recombination analyses were conducted against a reference dataset of HIV-1 group M subtypes (A, B, C, D, F, G, H, J and K) and CRF02_AG downloaded from the Los Alamos HIV Sequence Database (<http://www.hiv.lanl.gov>) as well as the top two CRF02_AG BLAST hits. (A) The upper left diagram in each scheme illustrates the genomic map, which was generated by the Recombinant HIV-1 Drawing Tool ([www.hiv.lanl.gov/content/sequence/DRAW_CRF/recom_mapper.html](http://www.hiv.lanl.gov/content/sequence/DRAW_CRF/recom_mapper.html)). The numbers above the diagram indicate the intersubtype recombination breakpoints in accordance with HXB2 numbering. The near full-length HIV-1 genome was divided into seven fragments based on the six recombination breakpoints, showing its unique mosaic structure. The subtype origin of each fragment is color coded in accord with informative analyses, and the color coding is defined in the middle of each scheme. The middle left diagram in each scheme displays the similarity plot analysis, in which the y-axis represents the percent similarity of the query sequence to the reference dataset. The bottom left diagram in each scheme displays the bootscan analysis, where the y-axis represents the bootstrap support value. The x-axes of both diagrams represent the nucleotide positions in accordance with HXB2 numbering. The dotted horizontal line specifies the 70% bootstrap support value, which was considered to be definitive for subtype origin. The color coding used for the similarity plot and bootscan analyses is identical to the color coding of the genomic map. The similarity plot and bootscan analyses were performed in SimPlot v3.5.1 software. The parameters included a sliding window of 400 nucleotides, overlapped by 40 nucleotides, with 1,000 bootstrap replicates. (B) The right diagram in each scheme illustrates the subregion confirmatory neighbor-joining tree analyses performed with MEGA X software. The neighbor-joining trees were constructed for each of the seven fragments characterized by the similarity plot and bootscan analyses. The phylogenetic analyses employed the Kimura two-parameter nucleotide substitution model with 1,000 bootstrap replicates to assess the reliability of the phylogenetic clustering results. A bootstrap support value of 70% was considered definitive for subtype origin. The region of the nucleotide sequences encoding each of the seven fragments is denoted above each tree with respect to HXB2 numbering. The dotted line ending with a black dot represents the query sequence of each tree. The color coding used for the neighbor-joining trees is identical to the color coding of the genomic map, similarity plot and bootscan analyses.


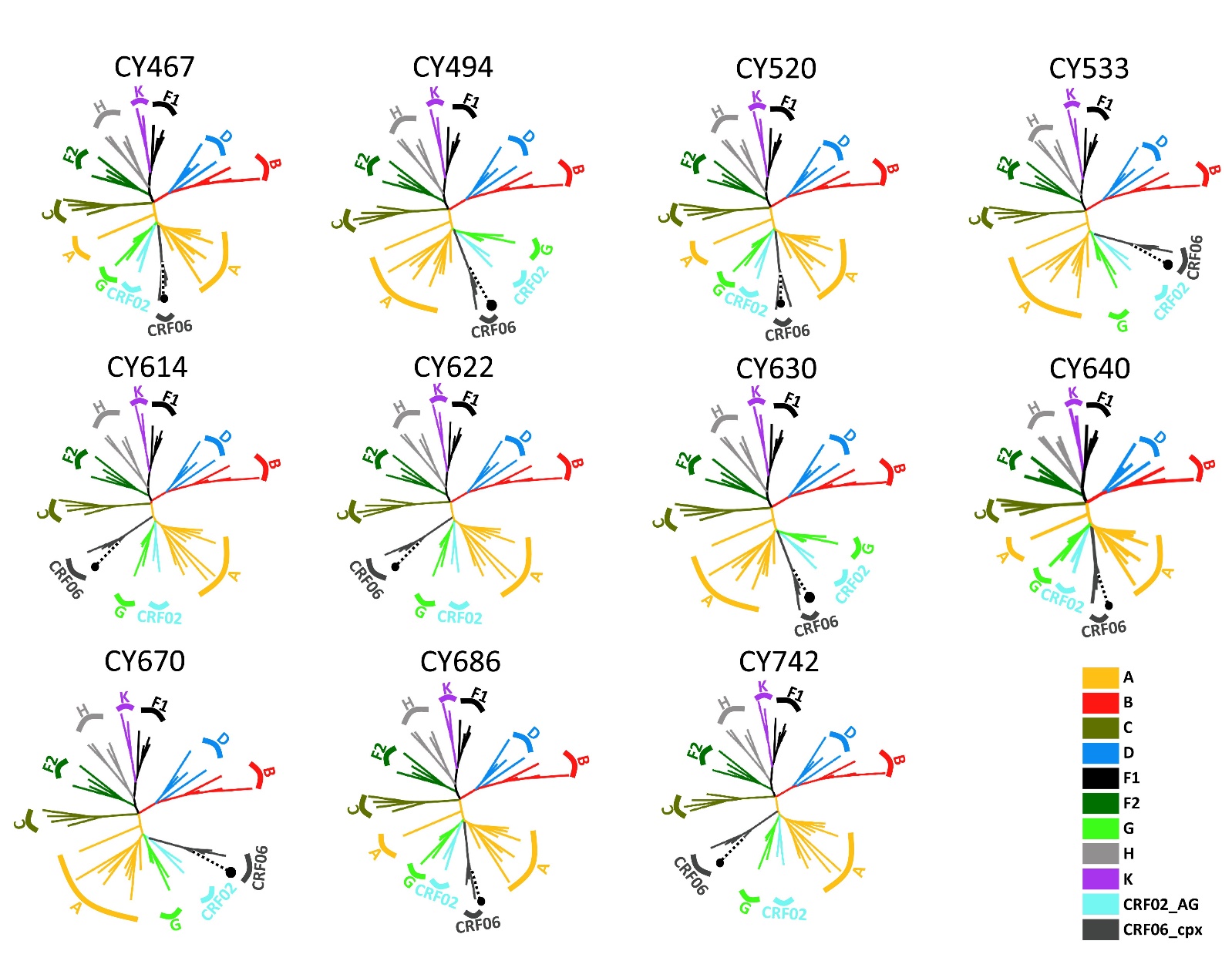


**Figure S2.** Subregion confirmatory neighbor-joining tree analyses of the last fragment (8434-8686 in the HXB2 genome) of each of the HIV-1 recombinant query sequences, as denoted above in each tree. The phylogenetic analyses was conducted in MEGA X software against a reference dataset of HIV-1 group M subtypes (A, B, C, D, F, G, H and K), downloaded from the Los Alamos HIV Sequence Database (<http://www.hiv.lanl.gov>) as well as the top three CRF06_cpx BLAST hits. Reference sequences for subtype J were removed from the reference dataset for these analyses. The phylogenetic analyses employed the Kimura two-parameter nucleotide substitution model with 1,000 bootstrap replicates to assess the reliability of the phylogenetic clustering results. A bootstrap support value of 70% was considered definitive for subtype origin. The dotted line ending with a black dot represents the query sequence of each tree. The neighbor-joining trees are color coded, and the color coding is defined at bottom right of the figure.


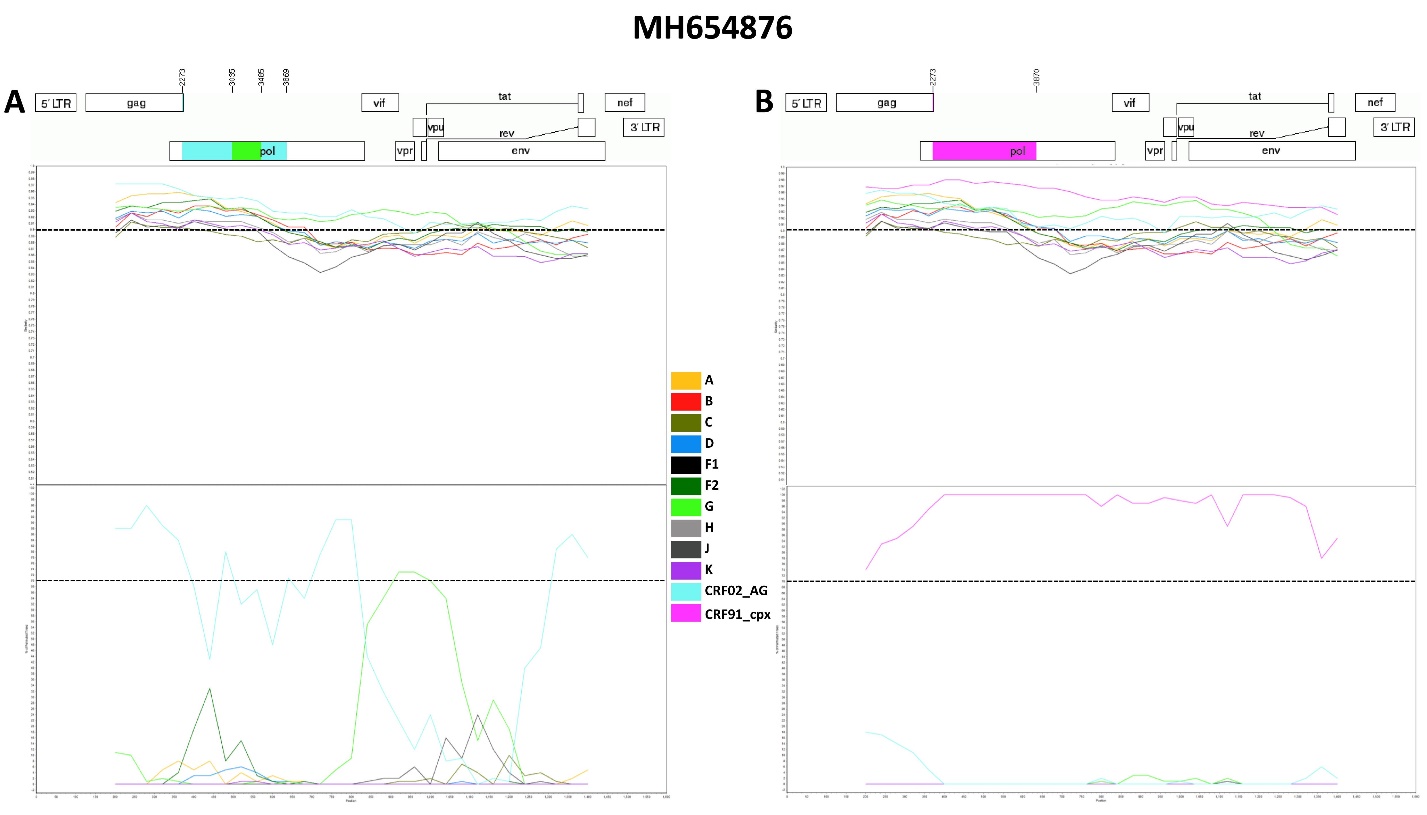

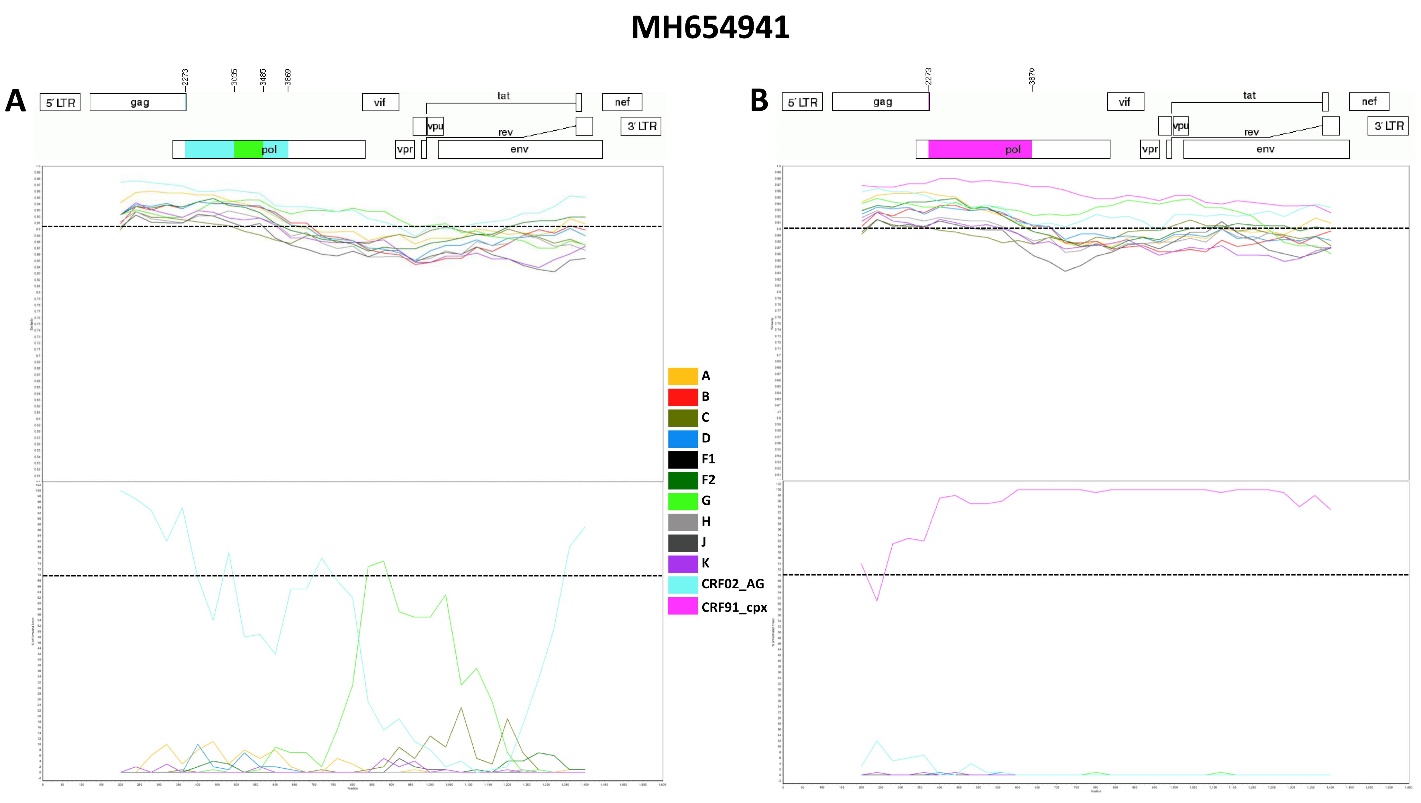


**Figure S3.** Recombination analyses of the two HIV-1 partial sequences (2273-3869 in the HXB2 genome), MH654876 and MH654941, which demonstrate the same mosaic structure as the CRF91_cpx strain. The above scheme represents the sequence MH654876, and the bottom scheme represents the sequence MH654941, as indicated above each scheme. (A) The upper left diagram in each scheme illustrates the genomic map, which was generated by the Recombinant HIV-1 Drawing Tool ([www.hiv.lanl.gov/content/sequence/DRAW_CRF/recom_mapper.html](http://www.hiv.lanl.gov/content/sequence/DRAW_CRF/recom_mapper.html)). The numbers above the diagram indicate the intersubtype recombination breakpoints in accordance with HXB2 numbering. The HIV-1 sequence was divided into three fragments based on the two recombination breakpoints presenting the same intersubtype mosaicism as the CRF91_cpx strain. The subtype origin of each fragment is color coded in accord with informative analyses, and the color coding is defined in the middle of both schemes. The middle left diagram in each scheme displays the similarity plot analysis, where the y-axis represents the percent similarity of the query sequence to the reference dataset. The bottom left diagram in each scheme displays the bootscan analysis, where the y-axis represents the bootstrap support value. The x-axes of both diagrams represent the nucleotide positions in accordance with HXB2 numbering. The dotted horizontal line specifies the 70% bootstrap support value, which was considered to be definitive for subtype origin. The color coding used for the similarity plot and bootscan analyses is identical to the color coding of the genomic map. The similarity plot and bootscan analyses were performed with SimPlot v3.5.1 software. The parameters included a sliding window of 400 nucleotides, overlapped by 40 nucleotides, with 1,000 bootstrap replicates. The recombination analyses, illustrated by the diagrams on the left of each scheme, were run against a reference dataset of HIV-1 group M subtypes (A, B, C, D, F, G, H, J and K) downloaded from the Los Alamos HIV Sequence Database (<http://www.hiv.lanl.gov>), and three BLAST hits for CRF02_AG. (B) The recombination analyses were repeated, as illustrated by the diagrams on the right of each scheme, using the aforementioned reference dataset along with the ten HIV-1 recombinant sequences.


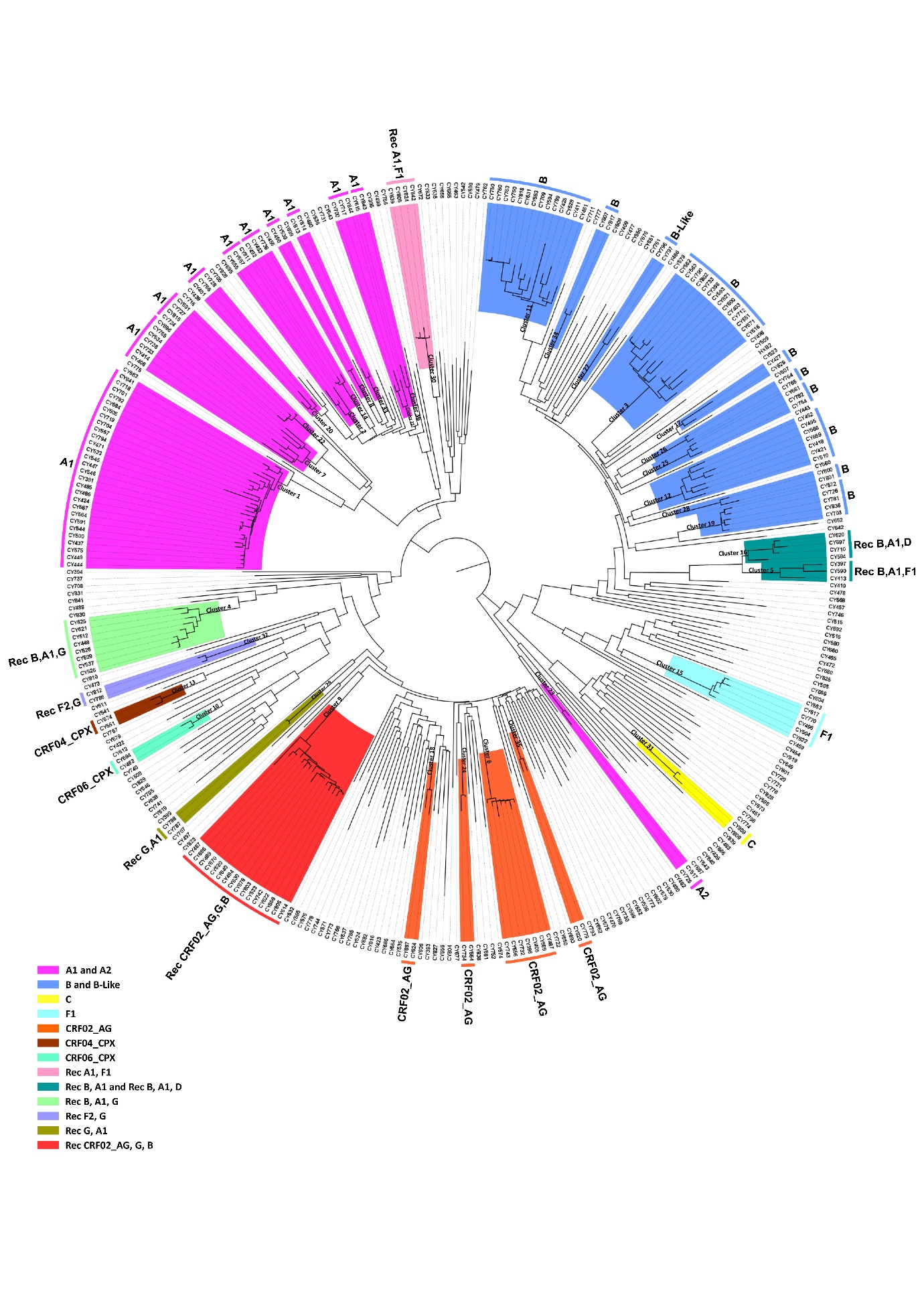


**Figure S4.** The most recent monthly maximum likelihood phylogenetic tree (September 2021) constructed as part of the near real-time surveillance monitoring system implemented by our laboratory website to provide up-to-date visualization analytics. This maximum likelihood phylogenetic tree consists of HIV-1 *pol* region (2253-5250 in the HXB2 genome) nucleotide sequences derived from all HIV-1-infected patients received by our laboratory from 9 March 2017 to 14 October 2021. The HIV-1 infected patients included as part of these monthly analyses are newly diagnosed or chronic. The patients are included in these analyses regardless of their treatment status. The HIV-1 phylogenetic clusters are highlighted by color coding based on the HIV-1 genotypic subtypes, and the color coding is explained below the tree. The HIV-1 genotypic subtypes determined by REGA-3.0 are indicated at the periphery of each phylogenetic cluster. Phylogenetic clusters were classified as transmission clusters on the condition of minimum of 3 patient samples clustering together. For the phylogenetic clustering analyses, parameters of 0.045 genetic distance as the threshold and 70% bootstrap support value were utilized.
